# CD146 Associates with Gp130 to Control a Macrophage Pro‐inflammatory Program That Regulates the Metabolic Response to Obesity

**DOI:** 10.1002/advs.202103719

**Published:** 2022-03-08

**Authors:** Hongxia Duan, Lin Jing, Jianquan Xiang, Chenhui Ju, Zhenzhen Wu, Jingyu Liu, Xinran Ma, Xuehui Chen, Zheng Liu, Jing Feng, Xiyun Yan

**Affiliations:** ^1^ Laboratory of Protein and Peptide Pharmaceutical Institute of Biophysics Chinese Academy of Sciences Beijing 100101 China; ^2^ College of Life Sciences University of Chinese Academy of Sciences 19A Yuquan Road Beijing 100049 China; ^3^ Joint Laboratory of Nanozymes in Zhengzhou University School of Basic Medical Sciences Zhengzhou University Zhengzhou 450001 China

**Keywords:** CD146, Gp130, macrophage polarization, obesity

## Abstract

The mechanism of obesity‐related metabolic dysfunction involves the development of systemic inflammation, largely mediated by macrophages. Switching of M1‐like adipose tissue macrophages (ATMs) to M2‐like ATMs, a population of macrophages associated with weight loss and insulin sensitivity, is considered a viable therapeutic strategy for obesity‐related metabolic syndrome. However, mechanisms for reestablishing the polarization of ATMs remain elusive. This study demonstrates that CD146^+^ ATMs accumulate in adipose tissue during diet‐induced obesity and are associated with increased body weight, systemic inflammation, and obesity‐induced insulin resistance. Inactivating the macrophage CD146 gene or antibody targeting of CD146 alleviates obesity‐related chronic inflammation and metabolic dysfunction. Macrophage CD146 interacts with Glycoprotein 130 (Gp130), the common subunit of the receptor signaling complex for the interleukin‐6 family of cytokines. CD146/Gp130 interaction promotes pro‐inflammatory polarization of ATMs by activating JNK signaling and inhibiting the activation of STAT3, a transcription factor for M2‐like polarization. Disruption of their interaction by anti‐CD146 antibody or interleukin‐6 steers ATMs toward anti‐inflammatory polarization, thus attenuating obesity‐induced chronic inflammation and metabolic dysfunction in mice. The results suggest that macrophage CD146 is an important determinant of pro‐inflammatory polarization and plays a pivotal role in obesity‐induced metabolic dysfunction. CD146 could constitute a novel therapeutic target for obesity complications.

## Graphical abstract

Under hyperlipidemia conditions, CD146 interacts with Gp130 to inhibit ACM (mainly IL‐6)‐induced STAT3 activation. It also activates the JNK signal and promotes the expression of M1‐related genes and SOCS3, which enhances the STAT3 inhibition. Deletion or blocking CD146 steers ATMs toward anti‐inflammatory polarization, thus attenuating obesity‐induced chronic inflammation and metabolic dysfunction.

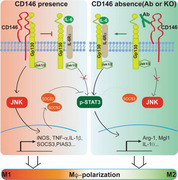

## 1 Introduction

Obesity and its associated comorbidities, type 2 diabetes and cardiovascular disease are life‐threatening chronic diseases.^[^ ^1^ ^]^ Compelling evidence suggests a causal role of chronic inflammation in obesity‐associated pathologic conditions, including liver damage and insulin resistance.^[^ ^2^ ^]^ Adipose tissue macrophages (ATMs), constituting about 5% of adipose stromal vascular cells or fractions (SVFs) in the lean state and up to 50% of SVFs during obesity,^[^ ^3^, ^4^ ^]^ have been recognized as key contributors to chronic inflammation.^[^ ^5^ ^]^ Obesity‐activated ATMs have complex phenotypes. Upon overnutrition, ATMs have been reported to switch from type 2 (M2; CD11c^−^CD206^+^) to type 1 (M1; CD11c^+^CD206^−^) polarization states, promoting local and systemic inflammation and insulin resistance.^[^ ^3^, ^6^, ^7^ ^]^ However, the identification of balanced M0 (CD206^−^CD11c^−^)^[^ ^8^ ^]^ and mixed (CD206^+^CD11c^+^)^[^ ^9^ ^]^ phenotypes indicates the existence of additional subtype diversity.^[^ ^10^, ^11^ ^]^ Targeting of maladaptive adipose tissue macrophage (ATM) phenotypes has the potential to restore the function of ATMs for the homeostasis of adipose tissue in obese individuals.

Recent studies suggest that glycoprotein 130 (Gp130) receptor ligands comprise potential therapeutic targets for obesity;^[^ ^12^, ^13^ ^]^ however, the role of Gp130 signaling in insulin resistance remains controversial.^[^ ^14^ ^]^ Gp130 is the common subunit of the receptor signaling complex for the interleukin‐6 (IL‐6) family of cytokines and is ubiquitously expressed.^[^ ^14^ ^]^ The effect of Gp130 ligands in metabolic disease is unclear and is likely to be context‐dependent, especially with respect to IL‐6. Although IL‐6 is generally considered a pro‐inflammatory cytokine, systemic ablation of IL‐6 in mice leads to mature‐onset obesity and insulin resistance.^[^ ^15^ ^]^ A recent study has shown that IL‐6 mediates anti‐inflammatory effects in macrophages by promoting higher expression of M2 phenotype‐associated genes.^[^ ^16^ ^]^ However, given the pro‐inflammatory capacity of IL‐6, it may not represent a promising therapeutic target for obesity treatment. Therefore, an ideal target should be more specific than IL‐6 but have similar anti‐inflammatory effects.

The cluster of differentiation 146 (CD146), an adhesion molecule, mediates tumor invasion and angiogenesis.^[^ ^17^ ^]^ Reportedly, its expression on T cells and macrophages^[^ ^18^, ^19^ ^]^ contributes to the development of non‐resolving inflammatory diseases, including multiple sclerosis,^[^ ^20^ ^]^ inflammatory bowel disease,^[^ ^21^ ^]^ cerebral malaria^[^ ^22^ ^]^ and atherosclerosis.^[^ ^19^ ^]^ Notably, elevated CD146 expression on macrophages under pro‐atherogenic hyperlipidemic conditions promotes foam cell formation and disrupts migration‐related signaling.^[^ ^19^ ^]^ Given the similar hyperlipidemic condition between atherosclerosis and obesity, in this study, we investigate the effects of CD146 on the ATM pro‐inflammatory capacity in a high‐fat diet (HFD)‐induced obesity mice model. Our findings suggest that under obesogenic conditions, CD146 is expressed on a subtype of ATMs and associates with Gp130 to maintain the M1‐like pro‐inflammatory phenotype and promotes the development of dietary obesity and adipose inflammation, leading to systemic insulin resistance. Conversely, inactivation of the CD146 gene or antibody targeting of CD146 releases the Gp130 protein from its association with CD146, steering ATMs toward M2‐like polarization, thus attenuating obesity‐induced inflammation and insulin resistance in mice. The study suggests the potential of macrophage CD146 as a novel and specific therapeutic target for obesity complications.

## 2 Results

### 2.1 CD146‐expressing Macrophages Accumulate in Adipose Tissue upon Diet‐Induced Obesity

**Figure 1:**
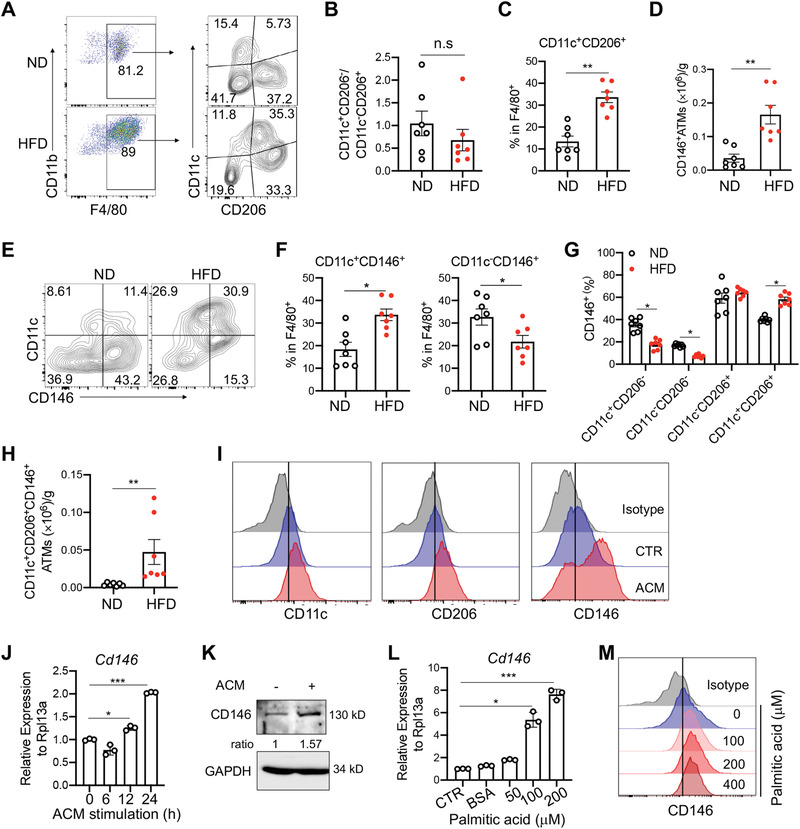
Accumulation of CD146‐expressing macrophages in adipose tissue during diet‐induced obesity. A) Surface staining of CD11b, F4/80, CD11c, and CD206 on CD45.1^+^CD11b^+^ leukocytes obtained from visceral adipose tissue (VAT) of mice fed with chow diet (ND) or high fat diet (HFD) (*n* = 7). The numbers in the outlined areas indicate the percentages of each gate. B) The ratio of percentages of CD11c^+^CD206^−^ to CD11c^−^CD206^+^ adipose tissue macrophages (ATMs) in ND and HFD‐fed mice (*n* = 7). C) Percentages of the CD11c^+^CD206^+^ population in F4/80^+^ macrophages (*n* = 7). D) Cell number of CD146^+^ ATMs in VAT from ND‐ and HFD‐fed mice (*n* = 7). E) Cell surface staining of CD11c and CD146 in VAT CD11b^+^F4/80^+^ cells from ND‐ and HFD‐fed mice. The numbers in the outlined areas indicate the percentages for each gate. F) Percentages of CD11c^+^CD146^+^ and CD11c^−^CD146^+^ populations in VAT F4/80^+^ macrophages from ND‐ and HFD‐fed mice (*n* = 7). G) Percentages of CD146^+^ cells in CD11c^+^CD206^−^, CD11c^−^CD206^−^, CD11c^−^CD206^+^, and CD11c^+^CD206^+^ populations from VAT macrophages in ND‐ and HFD‐fed mice (*n* = 7). H) Cell numbers of CD11c^+^CD206^+^CD146^+^ ATMs in VAT from ND‐ and HFD‐fed mice (*n* = 7). I) Surface staining of CD11c, CD206 and CD146 in adipocyte culture medium (ACM) or control medium‐treated bone marrow‐derived macrophages (BMDMs; representative of *n* = 3). J) Relative CD146 mRNA expression in BMDMs after ACM stimulation for the indicated times (*n* = 3). Data were normalized to that of no ACM treated group. K) Immunoblotting of CD146 protein in BMDMs with or without ACM stimulation (representative of *n* = 3). L) Relative CD146 mRNA expression in BMDMs after stimulation with the indicated concentrations of palmitic acid for 24 h. Data were normalized to that of the no palmitic acid‐treated group. M) Flow cytometry analysis of CD146 protein expression on BMDMs after stimulation with the indicated concentration of palmitic acid for 24 h. Each symbol represents an individual mouse (B, C, D, F, G, and H) or an experiment (J, L); the short horizontal lines indicate the mean ± SEM (B, C, D, F, G, and H) or SD (J, L). Significance analysis was performed using a two‐tailed *t*‐test (B, C, D, F, and H) or one‐way ANOVA (G, J, and L). *n*.s., not significant; *p < 0.05, **p < 0.01, ***p < 0.001.

To investigate whether macrophage CD146 is involved in the development of obesity, we first performed flow cytometry to evaluate CD146^+^ macrophages in visceral adipose tissue (VAT) of C57BL/6 mice fed standard chow (ND) or HFD (60% kcal) for 12 weeks (Figure S1A, Supporting Information). Consistent with previous reports,^[^ ^7^ ^]^ both the number and percent of CD11b^+^F4/80^+^CD11c^+^ macrophages were significantly higher in VAT from HFD compared with ND mice (Figure S1B–D, Supporting Information). Interestingly, the ratio of CD11c^+^CD206^−^ to CD11c^−^CD206^+^ cells among F4/80^+^ macrophages between ND and HFD mice did not differ significantly (**Figure** **1**A,B); however, the percent of CD11c^+^CD206^+^ cells was notably increased upon diet‐induced obesity (Figure 1C). To clarify the function of CD146^+^ macrophages, we evaluated the number and percent of CD146^+^ cells in CD11c^+^ and/or CD206^+^ cell populations. As shown in Figure 1D, the number of CD146^+^ macrophages was increased in VAT of HFD‐fed mice. Furthermore, subtype analysis revealed that the percentage of CD11c^+^CD146^+^ macrophages was significantly increased, while the percentage of CD11c^−^CD146^+^ macrophages was reduced (Figure 1E,F). Additionally, the percentage of CD146‐positive cells among the CD11c^+^CD206^−^ and CD11c^−^CD206^−^ cell populations was significantly decreased in HFD‐fed mice (Figure 1G). ≈60% of CD206^+^ macrophages were CD146‐positive, regardless of whether they were from ND or HFD‐fed mice, though both the percentage and number of CD146^+^ cells in the CD11c^+^CD206^+^ cell population were significantly increased in HFD‐fed mice (Figure 1G,H). These data suggest that the CD11c^+^CD206^+^CD146^+^ subpopulation in VAT of HFD‐fed mice is over‐represented during obesity.

To further evaluate the obesity‐associated expression of CD146 on macrophages, we measured CD146 expression on mouse bone marrow‐derived macrophages (BMDMs) treated with adipocyte culture medium (ACM), which contains a large amount of lipid and cytokine components, and well mimics the microenvironment of adipose tissue.^[^ ^23^, ^24^ ^]^ Compared with BMDMs treated with medium control, ACM‐treated BMDMs showed higher levels of CD11c, CD206, and CD146 by flow cytometry analysis (Figure 1I). Real‐time PCR and western blotting analyses also revealed that ACM treatment significantly increased the mRNA and protein levels of CD146 (Figure 1J,K). To confirm that CD146 expression is regulated by saturated fatty acids, we treated BMDMs with palmitic acid, which mediated a dose‐dependent increase in CD146 expression (Figure 1L,M). Together, these data suggest that saturated fatty acids promote the expression of CD146 on macrophages and that CD146^+^ macrophages represent a potential pro‐inflammatory population in obesity.

### 2.2 Deficiency of Macrophage CD146 Prevents Diet‐induced Obesity

**Figure 2:**
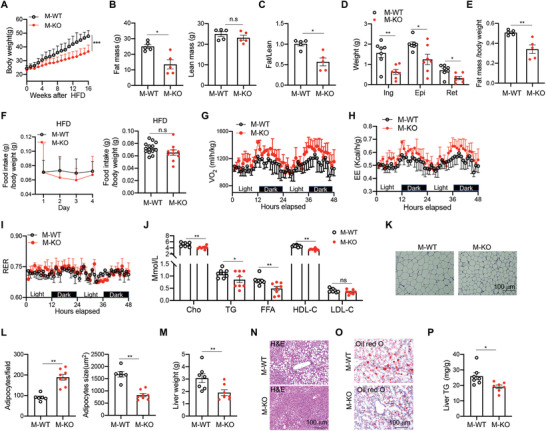
Deficiency of CD146 on macrophages prevents diet‐induced obesity. A) Weight gains of CD146‐conditional knockout mice (M‐KO) and their control littermates (M‐WT) fed with HFD (*n* = 8 for each group). B) EchoMRI scans of fat mass (left) and lean mass (right) of M‐WT and M‐KO mice fed with HFD for 12 weeks (*n* = 5). C) The ratio of fat mass to lean mass (*n* = 5). D) Weights of inguinal white adipose tissue (ingWAT), epididymal white adipose tissue (epiWAT), and retroperitoneal white adipose tissue (retWAT) from M‐WT and M‐KO mice (*n* = 7). E) The ratio of fat mass to body weight in M‐WT and M‐KO mice fed with HFD (*n* = 5). F) The relative daily food intake and average food intake of HFD‐fed mice (*n* = 14 for M‐WT or 10 for M‐KO). The metabolic‐cage analysis for G) the volume of oxygen consumption (VO2), H) energy expenditure (EE), and I) respiratory exchange ratio (RER) (*n* = 5 for each group). J) The concentrations of sera lipid metabolic parameters in M‐WT and M‐KO mice fed with HFD (*n* = 8). K) Representative H&E stained images of WAT (*n* = 3 images per mouse). L) Numbers of adipocytes per field (left) or areas of adipocytes (right) (*n* = 6 for M‐WT or 8 for M‐KO mice). M) Liver weight of HFD‐fed M‐WT and M‐KO mice (*n* = 7). N) Representative H&E stained images of the liver (*n* = 3 images per mouse). O) Representative Oil‐red‐O stained images of the liver (*n* = 3 images per mouse). P) Liver triglyceride (TG) of HFD‐fed M‐WT and M‐KO mice (*n* = 7). Each symbol (B, C, D, E, F, J, L, M, and P) represents an individual mouse; the short horizontal lines indicate the mean ± SEM. For significance analysis, a two‐tailed t‐test (B, C, E, F, L, M, and P) or one‐way ANOVA (J) was performed. *n*.s., not significant; *p < 0.05, **p < 0.01.

To further evaluate the role of CD146^+^ macrophages in diet‐induced obesity, we applied the HFD model to macrophage CD146‐conditional knockout mice (M‐KO) and their control littermates (M‐WT). The mouse strains had similar weight gain with regular chow diets (Figure S2A, Supporting Information). However, after 16 weeks, the weight gain was significantly lower in HFD‐fed M‐KO mice than that in HFD‐fed M‐WT mice, suggesting that CD146^+^ macrophages promote diet‐induced obesity (**Figure** **2**A). Subsequent EchoMRI scans and adipose tissue analysis confirmed that the lower weight in M‐KO mice stemmed from fat mass decrease without any change in lean mass (Figure 2B–E). Notably, the reduced weight gain in HFD‐fed M‐KO mice compared with HFD‐fed M‐WT mice was not associated with food intake (Figure 2F), suggesting that the differences could be metabolic in nature. To confirm this possibility, we performed a metabolic‐cage analysis. As shown in Figure S2B–E (Supporting information), mice fed with a regular chow diet had similar food intake, energy expenditure, and respiratory‐exchange ratio (RER). In contrast, HFD‐fed M‐KO mice displayed greater energy expenditure than their WT control littermates (Figure 2G,H); however, no difference was detected in their RER and physical activities (Figure 2I and Figure S2F, Supporting Information), suggesting that CD146^+^ macrophages are associated with obesity‐induced metabolic changes.

To further elucidate the contribution of CD146^+^ macrophages to obesity, we evaluated sera lipid metabolic parameters. M‐KO mice had reduced serum levels of cholesterol (Cho), triglyceride (TG), free fatty acids (FFA), high‐density lipoprotein (HDL) and low‐density lipoprotein (LDL) (Figure 2J). Hematoxylin and eosin (H&E) staining of VAT revealed markedly smaller adipocytes in M‐KO mice compared with M‐WT mice (Figure 2K), which is consistent with the reported association of hypertrophic visceral adipocytes with increased FFA levels.^[^ ^25^ ^]^ Furthermore, the number of adipocytes per field was also increased in M‐KO mice than in the M‐WT mice (Figure 2L). It has been shown that hypertrophic adipocytes are more lipolytic and lead to an augmented risk of fatty liver.^[^ ^25^, ^26^ ^]^ Therefore, we isolated livers from M‐WT and M‐KO mice and measured their weights and lipid content. M‐KO mice had smaller and lighter livers (Figure 2M), with smaller clusters of lipid droplets (Figure 2N,O), which was confirmed by measuring the liver TG (Figure 2P). Collectively, these data suggest that CD146^+^ macrophages promote the development of diet‐induced obesity.

### 2.3 Deficiency of Macrophage CD146 Improves Insulin Sensitivity

**Figure 3:**
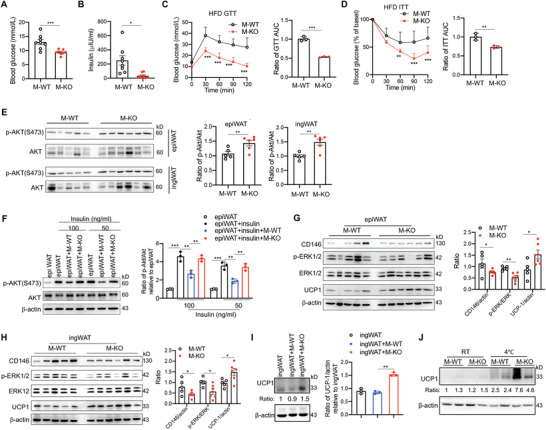
Deficiency of CD146 on macrophages improves insulin sensitivity. Fasting blood concentrations of A) glucose and B) insulin in HFD‐fed M‐WT and M‐KO mice. C) Oral glucose tolerance test (GTT) in M‐WT and M‐KO (*n* = 8 for each group) mice fed an HFD for 12 weeks. Right, the ratio of the area under the curve (AUC). D) Insulin tolerance test (ITT) in HFD‐fed M‐WT and M‐KO adjusted to the percentage of fat mass gained after 12 (M‐WT) or 20 (M‐KO) weeks (*n* = 7). Right, the ratio of the AUC. E) M‐WT (*n* = 5) and M‐KO (*n* = 6) HFD‐fed mice were injected intraperitoneally with 1 U kg^−1^ insulin. Immunoblot analyses of epiWAT and ingWAT tissue samples were performed using antibodies specific to AKT and phospho‐AKT (pAKT‐S473). Right histogram panels, quantification of p‐Akt levels relative to total Akt levels in epidermal white adipose tissue (epiWAT) and inguinal white adipose tissue (ingWAT). F) Ex vivo analysis of the insulin sensitivity of epiWAT cocultured with M‐WT or M‐KO macrophages (representative of *n* = 3). Immunoblot analyses of G) epiWAT and H) ingWAT tissue samples using antibodies specific to CD146, ERK, phospho‐ERK, UCP1, and *β*‐actin. Right histogram panels, quantifications of CD146 levels relative to *β*‐actin, p‐ERK levels to ERK, and UCP1 levels to *β*‐actin. I) Immunoblot analyses of ingWAT tissue samples cocultured with or without M‐WT or M‐KO macrophages for 24 h using antibodies specific to UCP1 and *β*‐actin (representative of *n* = 3). J) Immunoblot analyses of UCP1 and *β*‐actin in ingWAT tissue samples from mice kept at room temperature (RT) or 4 ℃ for 72 h (representative of *n* = 3). Each symbol represents an individual mouse (A, B, E, G, and H) or an experiment (C, D, F, and I); the short horizontal lines indicate the mean ± SEM (A, B, E, G, and H) or SD (C, D, F, and I). For significance analysis, a two‐tailed t‐test (A, B, C, D, and E) or two‐way ANOVA with multiple‐comparison test (C and D) or one‐way ANOVA (F, G, H, and I) was performed. *p < 0.05, **p < 0.01, ***p < 0.001.

Obesity is associated with glucose tolerance dysfunction and insulin resistance. Therefore, we examined the effect of CD146^+^ macrophages on glucose homeostasis. Fasting glycemia was significantly reduced in HFD‐fed M‐KO mice after 16 weeks (**Figure** **3**A), with a corresponding reduction in fasting insulinemia (Figure 3B). Furthermore, in glucose tolerance tests (GTTs), HFD‐fed M‐KO mice had improved glucose tolerance (Figure 3C). These data suggest that CD146^+^ macrophages disrupt glucose homeostasis. To validate this speculation, we performed insulin tolerance tests (ITTs). The M‐KO mice were more responsive than their M‐WT littermates to insulin stimulation (Figure 3D). These genotype‐specific metabolic differences are likely related to weight gain, because no significant differences in glucose tolerance and insulin sensitivity between ND‐fed M‐WT and M‐KO mice were observed (Figure S3A–C, Supporting Information). As additional confirmation, we isolated epidermal and inguinal white adipose tissue (epiWAT and ingWAT) from HFD‐fed mice treated with insulin for 10 min. As shown in Figure 3E, AKT phosphorylation levels in epiWAT and ingWAT were higher in M‐KO mice than in M‐WT mice. The enhanced AKT activation in M‐KO mice was confirmed in skeletal muscle and liver tissue (Figure S3D, Supporting Information). Together, these data suggest that insulin signaling under HFD conditions is more potent in M‐KO mice. To evaluate whether this difference in insulin signaling is determined by CD146^+^ macrophages, we cocultured WAT isolated from HFD‐fed WT mice with or without M‐WT or M‐KO macrophages. After insulin stimulation, the AKT phosphorylation levels were significantly increased in WAT cultured alone, while the phosphorylation was suppressed to about 60% in WAT cocultured with M‐WT macrophages. In contrast, coculture with M‐KO macrophages restored the phosphorylation level to about 90% (Figure 3F). These results confirm that CD146^+^ macrophages inhibit the insulin sensitivity of WAT.

Macrophages in VAT are associated with diminished beige adipogenesis mediated by ERK activation.^[^ ^27^ ^]^ Therefore, we evaluated the ERK phosphorylation and expression of uncoupling protein UCP1, which induces thermogenic activity in WAT. Both epiWAT and ingWAT from M‐KO mice had reduced ERK phosphorylation and elevated UCP1 levels (Figure 3G,H and Figure S3E, Supporting Information), suggesting that CD146 deletion in macrophages reduces the inflammation‐associated impairment of beige adipogenesis in obesity. The upregulation of UCP1 was further confirmed by ex vivo coculture with M‐WT or M‐KO macrophages (Figure 3I) and cold‐induced thermogenesis in mice (Figure 3J and Figure S3F, Supporting Information).

### 2.4 Deficiency of Macrophage CD146 Reduces their Retention in Adipose Tissues

**Figure 4:**
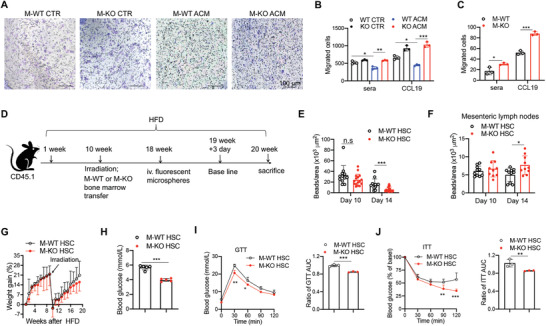
Macrophage deficiency of CD146 reduces macrophage retention in the adipose tissue. A) Represented images (200×) and B) statistical analysis of BMDMs treated with medium or ACM for 24 h (representative of *n* = 3). Migration assays were performed in the presence of sera or CCL19. C) Migrated cells from isolated VAT ATMs in the presence of sera or CCL19 (representative of *n* = 3). D) Schematic representation of the macrophage tracking model. E) In vivo analysis of the recruitment of bead‐labeled macrophages to and retention in VAT of CD146^M‐WT^→CD45.1^+^ (M‐WT HSC) and CD146^M‐KO^→CD45.1^+^ (M‐KO HSC) mice fed with HFD (*n* = 5 mice per group). F) Mean numbers of beads in mesenteric lymph nodes on days 10 and 14 (*n* = 5 mice per group). G) Body weight gain of M‐WT HSC and M‐KO HSC mice (*n* = 5). H) Fasting blood concentrations of glucose in M‐WT HSC and M‐KO HSC mice (*n* = 5). I) GTT and J) ITT of M‐WT HSC and M‐KO HSC mice (*n* = 5 for each group). The histograms on the right show the corresponding AUCs. Each symbol represents an experiment (B, C, I, and J) or an individual section (E, F) or mouse (H); the short horizontal lines indicate the mean ± SEM (H) or SD (B, C, E, F, I, and J) . For significance analysis, one‐way ANOVA (B, C, E, and F) or two‐tailed t‐test (H, I, and J) or two‐way ANOVA with multiple‐comparison test (I and J) was performed. *n*.s., not significant; *p < 0.05, **p < 0.01, ***p < 0.001.

The number of macrophages in adipose tissues was determined by the ratio of influx to egression. In our previous study, we demonstrated that CD146 inhibits the egression of macrophages from atheroma plaques during atherosclerosis.^[^ ^19^ ^]^ In this study, we observed that interleukin 4 (IL‐4)‐induced M2‐like macrophages have lower levels of CD146 expression (Figure S4A,B, Supporting Information) and higher migration ability (Figure S4C,D, Supporting Information) than lipopolysaccharide (LPS)/ interferon gamma (IFN‐*γ*)‐induced M1‐like macrophages. In addition, CD146‐deletion downregulated some M1/M2‐related membrane molecules, such as F4/80, major histocompatibility complex class II (MHCII), CD206, CD11c, and CD16/32 (Figure S4E, Supporting Information). Therefore, we hypothesized that the expression of CD146 may block the egression of ATMs from VAT. Consistent with this possibility, ACM treatment reduced the migration capacity of BMDMs, which was restored by CD146 deficiency; similar results were observed using the chemokine (C‐C motif) ligand 19 (CCL19) (**Figure** **4**A,B), which has been reported to mediate the emigration of tissue macrophages into draining lymph nodes.^[^ ^28^ ^]^ Next, we isolated ATMs from M‐WT and M‐KO mice fed with HFD for 12 weeks. As shown in Figure 4C, an increase in migration ability was observed for ATMs from M‐KO mice.

To confirm the role of CD146 on the emigration of ATM from VAT, we performed a macrophage tracking experiment with a bone marrow transfer model (Figure 4D).^[^ ^19^ ^]^ Bone marrow cells from M‐WT or M‐KO donor mice were transferred to lethally irradiated CD45.1^+^ CD146 WT recipient mice that had been fed with HFD for 10 weeks. The mice were injected with fluorescent microspheres when the reconstitution was complete (after 8 weeks). On the 10^th^ day after microsphere injection, when there was optimal recruitment of labeled monocytes into VAT and clearance of labeled monocytes from the peripheral blood (Figure S4F,G, Supporting Information), was defined as the baseline. The numbers of labeled macrophages in VAT were similar at baseline but were significantly reduced on day 14 in VAT from M‐KO→CD45.1 mice compared with M‐WT→CD45.1 mice (Figure 4E), with simultaneously increased numbers of labeled macrophages in mesenteric lymph nodes (Figure 4F). These results suggest that while CD146 inhibits the emigration of macrophages from the VAT to the periphery, it has little effect on the migration from the periphery to the VAT, which is consistent with our previous study.^[^ ^19^ ^]^ Moreover, although there was no significant difference in weight gain (with M‐KO→CD45.1 mice showing less weight gain in the last two weeks) (Figure 4G, Figure S4H, Supporting Information), lower fasting glycemia levels were detected in M‐KO→CD45.1 mice than in M‐WT→CD45.1 mice (Figure 4H). Importantly, M‐KO→CD45.1 transfer improved glucose tolerance and insulin sensitivity (Figure 4I,J). Collectively, these data suggest that CD146 on macrophages facilitates the retention of ATM and promotes inflammation in diet‐induced obesity.

### 2.5 Deficiency of Macrophage CD146 Mitigates Pro‐Inflammatory Polarization

**Figure 5:**
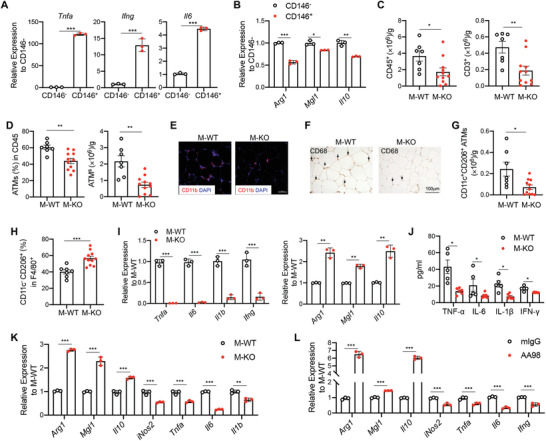
Macrophage deficiency of CD146 mitigates pro‐inflammatory polarization. Relative mRNA expression of A) M1‐ and B) M2‐like genes in CD146^+^ and CD146^−^ VAT macrophages isolated from HFD‐fed mice (*n* = 3). C) Cell numbers of CD45^+^ and CD3^+^ cells in VAT from M‐WT (*n* = 7) and M‐KO (*n* = 11) HFD‐fed mice. D) The percentages and numbers of CD11b^+^F4/80^+^ ATMs in VAT from M‐WT (*n* = 7) and M‐KO (*n* = 11) HFD‐fed mice. E) Immunofluorescent staining of CD11b (red) in VAT. Nuclei were counterstained with DAPI (blue) (representative of *n* = 3). Scale bar, 50 µm. F) Representative CD68‐stained images of VAT (representative of *n* = 3). G) Numbers of CD206^+^CD11c^+^ ATMs in VAT from M‐WT (*n* = 7) and M‐KO (*n* = 11) HFD‐fed mice. H) Percentages of CD11c^−^CD206^+^ cells in VAT ATMs from M‐WT (*n* = 7) and M‐KO (*n* = 11) HFD‐fed mice. I) Relative gene expression of *Tnfa*, *Il6*, *Il1b*, and *Ifng* in isolated ATMs from M‐WT and M‐KO HFD‐fed mice (*n* = 3). J) Concentrations of sera TNF‐*α*, IL‐6, IL‐1*β*, and IFN‐*γ* from M‐WT (*n* = 5) and M‐KO (*n* = 6) mice. K) Relative expression of M2‐related and M1‐related genes in the BMDMs after ACM stimulation (representative of *n* = 3). L) Relative expression of M2‐related and M1‐related genes in BMDMs after ACM stimulation in the presence of anti‐CD146 antibody AA98 or control isotype mouse IgG (50 µg mL^−1^) (representative of *n* = 3). Each symbol represents an individual mouse (A, B, C, D, G, H, I, and J) or an experiment (K and L); the short horizontal lines indicate the mean ± SEM (A, B, C, D, G, H, I, and J) or SD (K and L). Significance analyses were performed using a two‐tailed *t*‐test (A, C, D, G, and H) or one‐way ANOVA (B, I, J, K, and L). *p<0.05, **p<0.01, ***p<0.001.

In obesity, hypertrophic adipose tissue and insulin resistance have been attributed to heightened inflammation, caused mainly by ATM pro‐inflammatory polarization.^[^ ^2^ ^]^ These findings suggest that CD146 controls the pro‐inflammatory polarization of ATM. To determine whether CD146^+^ macrophages are pro‐inflammatory, we isolated CD146^+^ and CD146^−^ macrophages from F4/80^+^CD11c^+^CD206^+^ macrophages in HFD mice and measured the expression of M1‐like genes, including *Tnfa*, *Il6* and *Ifng*, and M2‐like genes, including *Arg1*, *Mgl1*, and *Il10*, using real‐time PCR. As shown in **Figure** **5**A and B, the expression of the M1‐like genes was higher, while those of M2‐like genes were lower in CD146^+^ cells than in CD146^−^ cells. These data suggest that CD146^+^ macrophages may constitute a pro‐inflammatory population during diet‐induced obesity. To confirm, we evaluated the inflammation levels and ATM phenotypes in VAT from M‐WT and M‐KO mice fed with HFD by flow cytometry and immunohistochemical staining. The total numbers of CD45^+^ cells, CD3^+^ T cells, and ATMs were significantly reduced in epiWAT obtained from M‐KO mice (Figure 5C–F). Moreover, the number of CD11c^+^CD206^+^ macrophages was significantly reduced in epiWAT from M‐KO mice (Figure 5G). Furthermore, among cells expressing the M2 marker CD206, there was a significant increase in CD11c^−^CD206^+^ macrophages in M‐KO mice (Figure 5H). Importantly, we found that VAT ATMs isolated from M‐KO mice displayed a marked down‐regulation of pro‐inflammatory M1‐related genes, including *Tnfa, Il6, Il1b*, and *Ifng*, and an upregulation of M2‐related genes, such as *Arg1*, *Mgl1*, and *Il10* (Figure 5I). Consistently, the plasma levels of TNF‐*α*, IL‐6, IL‐1*β*, and IFN‐*γ*, measured using cytokine array analysis, were decreased in M‐KO mice (Figure 5J). These data suggest that CD146 deficiency polarizes macrophages toward an M2‐like phenotype during diet‐induced obesity.

To confirm these findings, we stimulated BMDMs from M‐WT and M‐KO mice with ACM over a time course and then detected M1‐ and M2‐related gene expression by real‐time PCR. As shown in Figure 5K, ACM‐treated M‐KO macrophages expressed higher levels of M2‐related genes, including *Arg1, Mgl1*, and *Il10*, and lower levels of M1‐related genes, including *iNos, Tnfa, Il1b*, and *Il6*. These results were further confirmed in palmitic acid‐stimulated BMDMs (Figure S5A, Supporting Information) and also in the LPS/IFN‐*γ* ‐induced M1 and IL‐4 induced M2 (Figure S5B, Supporting Information). As additional confirmation, inhibition of CD146 with a functional antibody, AA98, decreased the expression of M1‐related genes and promoted the expression of M2‐related genes (Figure 5L). Together, these results suggest that CD146 deletion promotes M2 polarization under hyperlipidemia conditions.

### 2.6 CD146 Interacts with Gp130 and Negatively Regulates the STAT3 Activation

**Figure 6:**
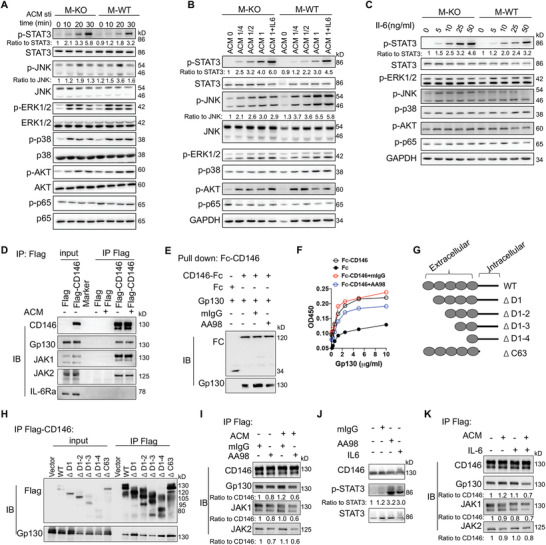
CD146 interacts with Gp130 and negatively regulates the STAT3 activation. Immunoblot analyses of BMDMs stimulated A) with ACM for the indicated times, or B) at the indicated concentrations, or C) with IL‐6 at the indicated concentrations using antibodies specific to ERK, JNK, p38, AKT, STAT3, and p65 and/or their phosphorylated forms (representative of *n* = 3). The ratio indicates quantification of the phosphorylated form relative to total protein. D) Immunoblot analysis (IB) of CD146, Gp130, JAK1, JAK2, and IL‐6R*α* in RAW264.7 cells transfected with CD146‐Flag plasmid or control Flag plasmid and stimulated with ACM followed by immunoprecipitation (IP) with Flag‐beads (representative of *n* = 3). E) Pull‐down assay of CD146‐Fc and Gp130 proteins in the presence of AA98 or control mouse IgG (mIgG) (representative of *n* = 3). F) Fc or Fc‐CD146^+^ with mIgG or AA98 was added to wells coated with different concentrations of Gp130, and ELISA was performed (representative of *n* = 3). G) Schematic representations of recombinant CD146 extracellular domains. H) Immunoblot analysis (IB) of Flag and Gp130 in 293T cells transfected with CD146‐Flag plasmid followed by IP with Flag‐beads (representative of *n* = 3). I) Immunoblot analysis (IB) of CD146, Gp130, JAK1, and JAK2 in RAW264.7 cells transfected with CD146‐Flag plasmid (RAW264.7‐CD146‐Flag) and treated with or without ACM plus mIgG or AA98 (50 µg mL^−1^) for 12 h (representative of *n* = 3). Ratios indicate quantification of the protein levels relative to CD146. Data are representative of at least three independent experiments. J) IB of p‐STAT3 in RAW264.7‐CD146‐Flag treated with or without ACM plus IL‐6 (100 ng mL^−1^), mIgG, or AA98 (50 µg mL^−1^) for 10 min (representative of *n* = 3). Ratios indicate quantification of the p‐STAT3 protein levels relative to STAT3. CD146 served as control. K) IB of CD146, Gp130, JAK1, and JAK2 in RAW264.7 cells transfected with CD146‐Flag plasmid and treated with or without ACM plus IL‐6 (100 ng mL^−1^) for 12 h (representative of *n* = 3). Ratios indicate quantification of the protein levels relative to CD146.

Our previous study showed that macrophage CD146 interacts with CD36 in response to oxidized low‐density lipoprotein (oxLDL) stimulation.^[^ ^19^ ^]^ However, this study revealed that CD36 expression is downregulated upon ACM stimulation (Figure S6A, B, Supporting Information), suggesting that the CD146‐CD36 interaction is not involved in macrophage polarization. To elucidate other potential mechanisms of the CD146‐promoted pro‐inflammatory program of macrophages in HFD‐induced obesity, we first evaluated signaling pathway activation in CD146 ^WT^ and CD146 ^KO^ macrophages after in vitro stimulation with ACM. As shown in **Figure** **6**A,B and Figure S6C, D (Supporting Information), stimulation for 10–30 min markedly increased the phosphorylation of ERK, JNK, p38, and AKT in CD146 ^WT^ cells, which is suggestive of increased pro‐inflammatory polarization, whereas p‐STAT3 or p‐p65 were marginally induced. However, STAT3 phosphorylation level was markedly higher, but JNK activation was lower in CD146 ^KO^ macrophages than in CD146 ^WT^ macrophages, though no significant difference was detected in ERK, p65, p38 and AKT phosphorylation in CD146 ^WT^ versus CD146 ^KO^ macrophages. To elucidate whether the M2‐like polarization in M‐KO cells depends on the STAT3 activation, we measured M1/M2‐related genes in STAT3 inhibitor‐treated BMDMs. As shown in Figure S6E (Supporting Information), the M2‐related genes (*Arg1, Mgl1*, and *Ym1*) were significantly increased in M‐KO cells; however, STAT3 inhibitor markedly reduced their upregulation. These data suggest that CD146‐induced pro‐inflammatory polarization occurs, at least in part, via JNK and STAT3 signaling pathways.

STAT3 signaling is downstream of the IL‐6 signaling pathway.^[^ ^29^ ^]^ Moreover, ACM is reported to typically contain IL‐6 cytokine. To clarify whether CD146 regulates IL‐6 signaling, we measured p‐STAT3 levels after stimulation by different doses of IL‐6. As shown in Figure 6B,C and Figure S6D (Supporting Information), the upregulation of p‐STAT3 levels was significantly increased in a dose‐dependent manner, either under ACM or IL‐6 stimulation. In addition, the p‐STAT3 levels were markedly higher in M‐KO cells than in M‐WT cells. These data suggest that CD146 is negatively involved in the activation of IL‐6‐induced signaling. To determine how CD146 controls IL‐6 signaling, we performed immunoprecipitation (IP) assays using a CD146‐Flag‐overexpressing RAW264.7 cell line. The results demonstrate that CD146 interacts with glycoprotein 130 (Gp130), a signal transducer for the IL‐6 family,^[^ ^13^ ^]^ and with Janus Kinase 1 and 2 (JAK1, JAK2), though interaction with IL‐6 receptor *α* (IL‐6R*α*) was not detected. The interaction between CD146, Gp130, and JAK1/2 was independent of IL‐6 but was slightly increased upon ACM stimulation (Figure 6D). In addition, no interaction of CD146 and IL‐6 was observed (Figure S6F, Supporting Information). These results indicated that Gp130 could be a new interacting partner protein for CD146 in macrophages. Pull‐down and ELISA assays verified that the interaction of CD146 and Gp130 is direct (Figure 6E,F). Therefore, to map which domain of CD146 is required for the interaction between CD146 and Gp130, we constructed several domain truncations of CD146, including domain 1 deletion (ΔD1), domain 1 to 2 deletion (ΔD1–2), domain 1 to 3 deletion (ΔD1–3), domain 1 to 4 deletion (ΔD1–4) and intracellular domain deletion (ΔC63) (Figure 6G). IP assays showed that these truncations did not affect the CD146/Gp130 interaction (Figure 6H), suggesting that the remaining domain (domain 5) may mediate this interaction. Furthermore, by using ELISA and IP assays, the CD146 antibody AA98, for which the binding epitope is located in domain 5,^[^ ^30^ ^]^ partially inhibited this interaction (Figure 6F,I). Consistent with a critical role for domain 5, AA98 treatment promoted the activation of STAT3 signaling (Figure 6J).

The Gp130 protein was not significantly increased in ATMs from HFD mice or ACM‐treated BMDMs (Figure S6G–I, Supporting Information). Because Gp130 is the co‐receptor for IL‐6 family members and IL‐6 promotes M2 polarization,^[^ ^13^, ^16^ ^]^ we determined whether IL‐6 blocks the association of CD146 and Gp130 in macrophages. As shown in Figure 6K, IL‐6 slightly affected the interaction between CD146 and Gp130 in the absence of ACM; however, in the presence of ACM, the interaction was blocked by IL‐6 to some extent. In addition, the activation of STAT3 induced by IL‐6 was similar to that observed with the AA98 treatment (Figure 6J). Because IL‐6 did not interact with CD146, these data suggest that CD146 could be a competitive inhibitor for the IL‐6 signal. Together, these data suggest CD146/Gp130 interaction blocks the IL‐6‐induced STAT3 signaling activation and promotes the pro‐inflammatory polarization of ATMs under hyperlipidemia conditions.

### 2.7 CD146/Gp130 Axis Promotes the SOCS3 Expression

**Figure 7:**
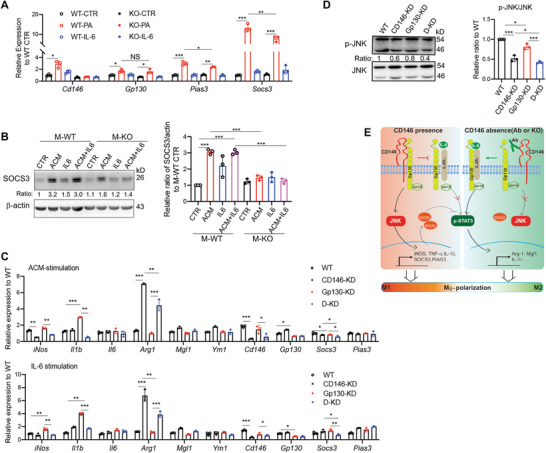
CD146/Gp130 axis promotes the SOCS3 expression. A) Relative gene expression in BMDMs stimulated with indicated stimuli (PA, 200 × 10^−6^ m; IL‐6, 50 ng mL^−1^) for 24 h (*n* = 3). B) Immunoblot analyses of SOCS3 protein in BMDMs treated with indicated stimuli for 48 h. Actin was served as control. Right panel, quantitative analysis of the SOCS3/actin ratio of BMDMs under various stimuli (*n* = 3). C) Analysis of mRNA levels from Raw264.7 cells. KD, knockdown; D‐KD, Gp130, and CD146 double knockdown. D) Immunoblot analyses of activated JNK protein in manipulated Raw264.7 cells treated with ACM for 20 min. KD, knockdown; D‐KD, Gp130 and CD146 double knockdown. Total JNK protein was served as the loading control. Right panel, quantitative analysis of the p‐JNK/JNK ratio (*n* = 3). E) Proposed model for the role of the CD146‐Gp130 interaction in macrophage polarization. Upon ACM stimulation, CD146 interacts with Gp130 to inhibit ACM (mainly IL‐6)‐induced STAT3 activation. It also activates the JNK signal and promotes the expression of M1‐related genes and SOCS3, which enhances the STAT3 inhibition. Conversely, when CD146 is blocked by an antibody or deleted, its association with Gp130 is disrupted; in turn, it inhibits the JNK signaling and activates STAT3 signaling, promoting the anti‐inflammatory polarization, which is similar to the effect of a larger amount of IL‐6‐stimulation alone. Each symbol represents an experiment (A–D); the short horizontal lines indicate the mean ± SD. One‐way ANOVA (A–D) was performed to test the statistical significance. *p < 0.05, **p < 0.01, ***p < 0.001.

The IL‐6 induced STAT3 activation was negatively regulated by other regulators, such as the well‐known negative regulator, Suppressor Of Cytokine Signaling 3 (SOCS3),^[^ ^31^ ^]^ and Protein Inhibitor Of Activated STAT 3 (PIAS3).^[^ ^32^ ^]^ To further elucidate how CD146 negatively regulates the STAT3 activation, we tested the above mentioned regulators in M‐WT and M‐KO BMDMs under hyperlipidemia conditions or IL‐6 stimulation. As shown in **Figure** **7**A, *Gp130* expression did not differ between M‐WT and M‐KO BMDMs; however, *Pias3* and *Socs3* genes were significantly reduced in M‐KO BMDMs. The downregulation of SOCS3 protein in M‐KO BMDMs was also confirmed by western blot (Figure 7B).

To further confirm the role of CD146‐Gp130 association in macrophage polarization, we knocked down Gp130 expression using siRNA in CD146‐knockdown Raw264.7 cells and evaluated the expression of polarization‐related genes, such as *Arg1, Mgl1, Ym1, iNOS, Il1b*, and *Il6*. As shown in Figure 7C, under either ACM or IL‐6 stimulation, Gp130 knockdown impaired the switch to M2‐polarization, while CD146 knockdown attenuated this defect. In addition, both Gp130 and CD146 knockdown impaired the SOCS3 expression to some extent, while Gp130 and CD146 double knockdown significantly reduced its expression (Figure 7C), suggesting that the SOCS3 expression at least partially depends on CD146/Gp130 signaling axis. Furthermore, the CD146/Gp130 signaling axis also played a positive role in JNK activation (Figure 7D), which has been reported to positively regulate the SOCS3 expression.^[^ ^33^ ^]^ Collectively, our findings suggest that CD146‐Gp130 interaction contributes to the pro‐inflammatory polarization of macrophages under hyperlipidemia conditions and that polarization is regulated by the balance between STAT3 and JNK signaling (Figure 7E, proposed model).

### 2.8 Antibody targeting of CD146 inhibits diet‐induced VAT inflammation and improves insulin resistance

**Figure 8:**
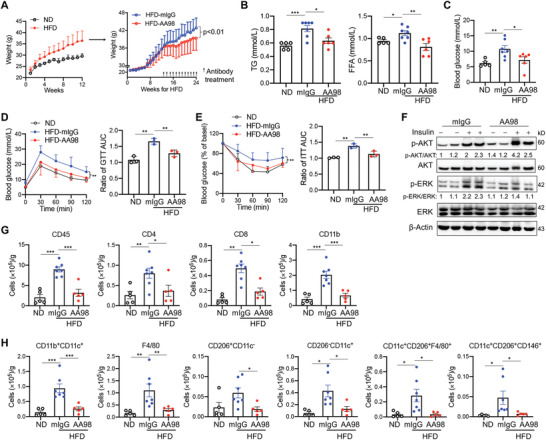
Targeting of CD146 with functional antibody AA98 inhibits VAT inflammation in diet‐induced obesity. A) Body weights of C57 mice fed with ND (*n* = 6) or HFD (*n* = 25). HFD mice were treated with AA98 antibody or control mIgG (*n* = 8 per group). Concentrations of B) sera TG and FAA and C) glucose in ND‐fed (*n* = 5) and HFD‐fed mice treated with AA98 (10 mg kg^−1^, *n* = 6) or mIgG (10 mg kg^−1^, *n* = 7) for 12 weeks. D) GTT and E) ITT of ND‐ and HFD‐fed mice treated with AA98 or mIgG (*n* = 6 each). The histograms on the right show the corresponding AUCs. F) Immunoblotting analysis of AKT, p‐AKT, ERK, p‐ERK, and *β*‐actin of VAT from antibody‐treated mice intraperitoneally injected with 1 U kg^−1^ insulin for 10 min (representative of *n* = 3). G,H) Cell numbers of infiltrated leukocytes in VAT from ND‐fed (*n* = 5) and antibody‐treated mice (*n* = 7 for mIgG or 5 for AA98 group). Each symbol represents an individual mouse (B, C, G, and H) or an experiment (D and E); the short horizontal lines indicate the mean ± SEM (B, C, G, and H) or SD (D and E). Two‐way ANOVA with multiple‐comparison test (A, D and E) or one‐way ANOVA (B, C, D, E, G, and H) was performed to test statistical significance. *p < 0.05, **p < 0.01, ***p < 0.001.

Our previous study demonstrates that AA98 alleviates atherosclerosis by promoting the emigration of macrophages from the atheroma.^[^ ^19^ ^]^ Therefore, we hypothesized that AA98 treatment might inhibit obesity‐associated inflammation and improve glucose homeostasis. To evaluate this possibility, we fed C57BL/6J male mice with HFD for 12 weeks (**Figure** **8**A). Then, we divided the mice into two groups and continued administering HFD. At the same time, we injected AA98 antibody or control mIgG (10 mg kg^−1^) intraperitoneally on the 13^th^ week and weekly for another 11 weeks (8 mice per group). The AA98‐treated mice had lesser gain weight (Figure 8B) and lower levels of sera TG and FFA than the control mIgG mice (Figure 8C). Moreover, the AA98 treatment significantly reduced fasting glycemia (Figure 8D). Though the effect of shorter treatment with AA98 antibody on glucose homeostasis was minimal, AA98 treatment modestly improved glucose tolerance and insulin resistance after 8 weeks (Figure S7A,B, Supporting Information), with significantly improved GTT and ITT results in AA98‐treated mice after 12 weeks (Figure 8E,F). These effects are unlikely to be explained by targeting CD146 on endothelial cells, as there were no significant differences in the weight gain, fasting glycemia, or glucose homeostasis between endothelial CD146 conditional knockout mice (CD146^EC‐KO^) and their controls after HFD feeding (Figure S7C–E, Supporting Information). In addition, higher insulin sensitivity of AA98‐treated mice was accompanied by a higher level of p‐AKT and a lower level of p‐ERK1/2 in VAT (Figure 8G). The VAT of AA98‐treated mice also had lower infiltration of CD45^+^ cells, including CD4^+^, CD8^+^, and CD11b^+^ populations than those in the VAT of the control mIgG mice (Figure 8H). Further analysis showed that CD11b^+^CD11c^+^ dendritic cells and CD11b^+^F4/80^+^ macrophages, especially CD11c^+^CD206^+^CD146^+^ macrophages, were significantly reduced after AA98‐treatment (Figure 8I). Collectively, these data suggest that macrophage CD146 could be a novel target for obesity‐induced chronic inflammation and insulin resistance.

## 3 Discussion

In this study, we identified a subset of CD146^+^ ATMs that accumulates in adipose tissue and promotes the development of dietary obesity and adipose inflammation, leading to systemic insulin resistance. We demonstrated an essential role for CD146 in macrophage polarization and the induction of a macrophage pro‐inflammatory program under hyperlipidemia conditions. Furthermore, we demonstrated that CD146 acts as a regulator of IL‐6 signaling by associating with Gp130. Inactivation of the CD146 gene or antibody targeting of CD146 releases the Gp130 protein from its association with CD146, steering ATMs toward anti‐inflammatory polarization, thus attenuating obesity‐associated adipose tissue inflammation and metabolic abnormalities, which is similar to the effect of IL‐6 on macrophage polarization. Collectively, this study provides evidence that macrophage CD146 targeting could serve as a potential therapeutic strategy to treat obesity‐induced insulin resistance.

Macrophages are the most important inflammatory cells in adipose tissues with key roles in obesity‐induced insulin resistance,^[^ ^10^ ^]^ and reestablishing disrupted macrophage polarization provides a basis for therapeutic targeting. During the obesity process, M1‐polarization is induced to promote pro‐inflammatory changes in adipose tissue, which may lead to systemic insulin resistance.^[^ ^34^ ^]^ Macrophage M2‐polarization is associated with less inflammation, lower body weight,^[^ ^35^, ^36^ ^]^ improved insulin sensitivity and white fat browning.^[^ ^27^, ^37^ ^]^ Targeting adipose tissue‐resident macrophages may serve as a potential antiobesity immunotherapy.^[^ ^38^, ^39^ ^]^ Our study indicates that CD146 is involved in the pro‐inflammatory polarization of macrophages under high‐fat conditions, which affects overall metabolic health. Under normal physiological conditions, the expression of CD146 in macrophages is low, while under inflammatory conditions, CD146 is significantly upregulated. Consistently, CD146 has been positively correlated with pulmonary inflammation.^[^ ^40^ ^]^ In addition, we found in a previous study that in atherosclerotic plaques, CD146 is highly expressed on macrophages, controlled by the oxLDL‐induced NF*κ*B signaling activation.^[^ ^19^ ^]^ Concordantly, in this study, we also observed an upregulation of CD146 expression on macrophages under high‐fat conditions. Reportedly, saturated fatty acid, such as palmitic acid, also activates the NF‐*κ*B signaling pathway;^[^ ^41^ ^]^ therefore, it was expected that this signaling pathway at least partially regulated CD146 expression during obesity. Taken together, these studies suggest that CD146 controls a macrophage pro‐inflammatory program for a variety of inflammatory conditions.

In obesity, M1‐like macrophages promote insulin resistance, which depends on FFA‐induced activation of inflammatory signaling pathways, including NF‐*κ*B and JNK. It has been shown that removing M1‐like macrophages from obese mice can restore insulin sensitivity^[^ ^10^ ^]^ without affecting body weight,^[^ ^42^ ^]^ suggesting that M1‐like macrophage‐induced insulin resistance is independent of body weight. In contrast, M2‐like macrophages promote insulin sensitivity and decrease body weight and total fat.^[^ ^35^, ^36^ ^]^ Therefore, strategies to increase the M2 proportion in obesity have potential therapeutic value. Here, we demonstrated that deletion of CD146 on macrophages reduces the expression of pro‐inflammatory genes, promotes insulin sensitivity, and notably reduces body weight. These data suggest that ablation of CD146 in macrophages not only reduces the expression of pro‐inflammatory factors but also promotes an M2‐like phenotype switch.

Although macrophages are implicated in systemic inflammation and metabolic dysfunction in obesity, previous studies have speculated that they do not play causal roles, given the lack of efficacy of anti‐inflammatory therapies in preventing obesity.^[^ ^39^, ^43^ ^]^ However, accumulating evidence demonstrates that macrophages are causal in energy storage and HFD‐induced weight gain.^[^ ^44^, ^45^ ^]^ A recent study has shown that adipose tissue‐resident macrophages promote lipid storage in mice through the production of platelet‐derived growth factor (PDGF), which induces lipid retention in WAT adipocytes in a paracrine manner.^[^ ^38^ ^]^ It introduces a new macrophage‐centered paradigm in metazoan energy storage. We determined that a population of CD146^+^ ATMs in adipose tissue plays a pro‐inflammatory role in diet‐induced obesity. In addition, this population promotes weight gain. Although the precise mechanism remains unclear, mice with deletion of this population showed increased UCP1 expression in ingWAT and more energy expenditure, which might be caused by the lower pro‐inflammatory factors (mainly TNF‐*α* and IL‐1*β*). We hypothesize that this population is composed of adipose tissue‐resident macrophages, given that in the bone marrow transfer experiment, though the insulin sensitivity was improved, the body weight gain was not significantly reduced in M‐KO HSC (CD146^M‐KO^→CD45.1^+^) transferred mice. Although we could not exclude the possible effect of other myeloid cells, such as neutrophils, our study identified a new population of adipose tissue‐resident macrophages in diet‐induced obesity.

CD11c^+^CD206^+^ macrophages were described as a novel macrophage subset to facilitate adipose tissue inflammation and insulin resistance in obese human subjects and mice models.^[^ ^9^, ^46^ ^]^ In mice, CD11c^+^CD206^+^ ATMs were markedly increased with the prolonged HFD supplementation.^[^ ^47^ ^]^ CD11c^+^CD206^+^ ATMs in humans were reported to have features of classically activated macrophages (M1), yet also have features of alternatively activated macrophages (M2),^[^ ^9^ ^]^ suggesting that CD11c^+^CD206^+^ ATMs have a dual role in adipose tissue. In our study, we identified that CD146^+^CD11c^+^CD206^+^ ATMs comprised ≈60% of total CD11c^+^CD206^+^ ATMs in HFD‐fed mice. In addition, this population was pro‐inflammatory and associated with obesity‐induced metabolic syndrome. Deleting CD146 or targeting CD146 with an antibody promoted the M2‐like phenotype and attenuated the obesity‐induced metabolic dysfunction. These results shed light on the possible novel anti‐inflammatory therapies to deal with the complications of obesity.

We also demonstrated that CD146 interacts with Gp130 in macrophages. Gp130 is a common subunit for receptors of IL‐6 family cytokines and mediates downstream signal transduction of the JAK/STAT, RAS/MAPK, and PI3K/AKT pathways.^[^ ^13^ ^]^ The activation of Gp130 signaling by IL‐6 family cytokines influences a wide range of important biological processes, including inflammation, cancer, and embryonic development.^[^ ^12^, ^14^, ^29^ ^]^ Myeloid cell‐specific deletion of Gp130 signaling leads to defective M2 macrophage polarization and exacerbates inflammatory response.^[^ ^48^ ^]^ In M2 polarization, STAT3 signaling is activated, while deletion of STAT3 promotes M1 polarization.^[^ ^49^, ^50^ ^]^ These studies suggest that Gp130‐STAT3 signaling facilitates M2 polarization and suppresses inflammation. On the contrary, blockade of Gp130 signaling has been reported to improve insulin sensitivity in diet‐induced obesity.^[^ ^13^ ^]^ Contradictory effects have also been observed in studies of IL‐6, which has been shown to increase in circulation with the degree of obesity.^[^ ^51^ ^]^ Injection of IL‐6 impairs insulin sensitivity in mice;^[^ ^52^ ^]^ however, global IL‐6 deficiency exacerbates insulin resistance and inflammation.^[^ ^15^ ^]^ Regulatory mechanisms that mediate diverse IL‐6 signaling effects remain elusive, though IL‐6 signaling in myeloid cells has been reported to attenuate obesity‐induced inflammation and insulin resistance by promoting alternative macrophage activation.^[^ ^16^ ^]^ This study demonstrated that CD146 is likely to be a key regulator of macrophage polarization that influences IL‐6 signaling. Under hyperlipidemic conditions, CD146 is likely to associate with Gp130 and/or other molecules, promoting M1‐like polarization. Blocking this association with *Cd146* gene deletion or CD146 antibody releases Gp130 and promotes IL‐6‐induced signaling, leading to M2‐like polarization. These data explain why macrophages in adipose tissue keep a pro‐inflammatory phenotype regardless of the presence of IL‐6. Given that agents targeting IL‐6 signaling have been used in global inflammation disorders,^[^ ^53^ ^]^ targeting CD146 to stimulate IL‐6 signaling in ATMs presents a promising alternative strategy for obesity and other metabolic syndromes that might be used to influence IL‐6 signaling effects.

Our data also highlight the importance of CD146 in macrophage retention during obesity‐induced insulin resistance. The accumulation of macrophages in obesity could be explained by several mechanisms, including recruitment‐dependent and independent mechanisms.^[^ ^10^ ^]^ Newer evidence suggests that recruitment‐independent mechanisms, such as impaired apoptosis, increased proliferation, and decreased egress, are important for adipose tissue inflammation. The egress of macrophages from inflamed tissue to local lymphoid tissues is mainly dependent on classical chemokines^[^ ^54^ ^]^ and neural guidance molecules.^[^ ^28^ ^]^ However, in obese VAT, the signals involved in macrophage egression and sustaining chronic inflammation remain largely unknown. A recent study revealed that upregulation of the neuroimmune guidance factor netrin‐1 facilitates the accumulation of macrophages in VAT by blocking chemokine‐induced migration, thus promoting insulin resistance,^[^ ^55^, ^56^ ^]^ which provides insight into the role of retention in the accrual of macrophages in obese VAT. Increasing evidence has shown that resident macrophages egress poorly from most tissues; therefore, it will be of interest in future studies to address how the egression of VAT macrophages impacts their total numbers and inflammatory potential and whether signals other than netrin‐1 are involved in this egression process.^[^ ^34^ ^]^ Our previous study showed that CD146 promotes macrophage retention in atherosclerotic plaques and accelerates atherosclerosis development.^[^ ^19^ ^]^ In this study, we further demonstrate that CD146 promotes the retention of macrophages in the obese state. Therefore, CD146 may serve as a potential therapeutic target for macrophage retention in a variety of inflammatory diseases.

CD146 has also been reported to function in association with CD36, a multifunctional class B scavenger receptor.^[^ ^19^ ^]^ CD36 is highly expressed on macrophages and adipocytes, implicating its role in the development of insulin resistance and diabetes.^[^ ^57^, ^58^ ^]^ CD36 functions as a principal receptor for the uptake of long‐chain fatty acids and oxidized lipids, thereby leading to lipid loading and foam cell formation, and its internalization triggers inflammatory signaling cascades. CD36 has been shown to facilitate macrophage infiltration and contribute to the inflammation in obese VAT; however, CD36 deficiency is associated with human insulin resistance syndrome.^[^ ^59^, ^60^ ^]^ Furthermore, in macrophages, the CD36 protein level has been shown to be reduced under high FFA conditions due to ubiquitination‐mediated degradation.^[^ ^61^ ^]^ In our previous study, we have shown that macrophage CD146 interacts with CD36 in response to oxLDL stimulation.^[^ ^19^ ^]^ However, ACM stimulation does not facilitate the interaction of CD146 and CD36. Moreover, CD36 expression is downregulated by ACM stimulation, suggesting that the CD146‐CD36 interaction is not involved in macrophage polarization. Nevertheless, we cannot exclude the possibility that the CD146‐CD36 axis might be involved in macrophages function in vivo. Therefore, the exact role of CD36 in obesity‐induced insulin resistance needs further study.

Our recent study also showed adipocytes CD146 as a receptor for angiopoietin‐like protein 2 (ANGPTL2) to promote adipogenesis and adipose inflammation.^[^ ^62^ ^]^ In macrophages, CD146 interacted with Gp130 but not with ANGPTL2, suggesting that CD146 plays complex and multiple roles in diet‐induced obesity. Indeed, increasing evidence demonstrates that CD146 is a multi‐functional molecule implicated in a variety of biological and pathological processes, such as tumor,^[^ ^63^ ^]^ inflammation,^[^ ^19^ ^]^ pathogenic infections,^[^ ^64^ ^]^ and autoimmune disease.^[^ ^18^, ^20^ ^]^ CD146 has been a promising candidate therapeutic target for various pathological processes. In obesity, CD146 is expressed on the vascular cells, macrophages and adipocytes, all of which play positive roles in developing adipose tissue inflammation and insulin resistance. Therefore, targeting CD146 could achieve the goal of “one stone kills three birds” in treating obesity and its associated comorbidities. In addition, for obesity‐non‐associated insulin resistance,^[^ ^1^ ^]^ a lean of insulin‐resistance macrophage CD146 would also be a promising therapeutic target.

## 4 Conclusion

In conclusion, our study provides compelling evidence that CD146 controls the pro‐inflammatory program by associating with Gp130 in diet‐induced obesity and therefore induces obesity‐induced insulin resistance. Our study explores not only a new mechanism for macrophage polarization in obesity‐induced inflammation and insulin resistance but also an effective therapeutic target for obesity‐induced metabolic syndrome.

## 5 Experimental Section

### Antibodies and Reagents

In this study, the anti‐CD146 antibody AA1, generated in the library,^[^ ^65^ ^]^ was used for immunoprecipitation, immunoblotting and flow cytometry, PE/Cy7‐conjugated rat antimouse CD146 (clone: ME‐9F1, Cat.134714, Biolegend) was used for flow cytometry, and anti‐CD146 (Cat.ab75769) was used for immunoblotting. In addition, the following other antibodies and reagents were also used: RBC Lysis Buffer (10X) (Cat.420301), Alexa Fluor 700 antimouse CD45.2 (Cat. 109822), FITC antimouse CD206 (MMR) (Cat. 141703), FITC antimouse/human CD11b (Cat. 101206), PE antimouse CD130 (gp130) (Cat. 149403), PerCP/Cyanine5.5 antimouse I‐A/I‐E(Cat. 107625), Brilliant Violet 711 antimouse CD16/32(Cat. 101337), and APC antimouse CD11c (Cat. 117310) were obtained from Biolegend. FcR Mouse Blocking Reagent (Cat. 130‐092‐575) was purchased from Miltenyi. Anti‐Mouse F4/80 Antigen eFluor 450 (Cat. 48‐4801‐82), anti‐Mouse CD11b PerCP‐Cyanine5.5 (Cat. 45‐0112‐82), and Accutase Enzyme Cell Detachment Medium (Cat. 00‐4555‐56) were obtained from eBioscience. Mouse (G3A1) mAb IgG1 Isotype Control (Cat.5415S) and antibodies against phospho‐p44/42 MAPK (ERK1/2) (Thr202/Tyr204) (Cat.4370S), p38*α* MAPK (L53F8) (Cat.9228S), Gp130 (Cat.3732S), phospho‐Akt (Ser473) (Cat.4060T), phospho‐NF‐*κ*B p65 (Cat.3033S), SAPK/JNK (Cat.9252S), Akt (pan) (40D4) (Cat.2920S), p44/42 MAPK (ERK1/2)(Cat.4695S), and NF‐*κ*B p65 (L8F6) (Cat.6956S) were purchased from GST. Gp130 antibody (Cat.ab226346), UCP1 antibody (Cat.ab10983) and SOCS3 (Cat.ab280884) were purchased from Abcam. IL‐6R antibody (A1570), JAK1 antibody (A18323) and JAK2 antibody (A19629) were purchased from ABclonal. Murine IL‐6, murine IL‐4, and murine IFN‐*γ* were obtained from PeproTech. Palmitic acid and 3X FLAG(R) peptide were purchased from Sigma. Mouse Insulin ELISA kits (Cat.ELM‐Insulin‐1) were from RayBiotech. STAT3 inhibitor (Cat.M3032) was purchased from Abmole.

### Mice

All animal experiments were performed in compliance with the guidelines for the care and use of laboratory animals and were approved by the institutional biomedical research ethics committee of the Institute of Biophysics, Chinese Academy of Sciences (permit number: SYXK2018‐45). All mice were housed in a pathogen‐free facility. All applicable institutional and/or national guidelines for the care and use of animals were followed.

Male C57BL/6J mice were obtained from the Department of Laboratory Animal Science, Peking University Health Science Center. Ly5.1 (CD45.1^+^) mice were obtained from the Nanjing Biomedical Research Institute of Nanjing University. Macrophage‐specific CD146 knockout mice (CD146^M‐KO^, Lyz2^cre/+^CD146^flox/flox^) and their control littermates (CD146^M‐WT^, Lyz2^cre/+^CD146^+/+^, or Lyz2^+/+^CD146^flox/flox^) were generated as described previously^[^ ^19^ ^]^ and were maintained in a pathogen‐free facility in the animal center of the Institute of Biophysics.

High‐fat diet (research diet, 60% fat) was initiated at 8 weeks of age and continued for 12–18 weeks with ad libitum access to water and food. The weights of mice were recorded weekly. The HFD‐induced obesity experiments were repeated independently at least three times.

### Metabolic Phenotype Measurement

Mice were fasted overnight, and the fasting blood glucose and insulin levels were determined using a glucose meter (Roche) an ELISA kit (RayBio), respectively. The GTT was performed by i.p. injecting D‐glucose (2 g kg^−1^ body weight) after overnight fasting. The ITT was performed by i.p. injecting insulin (0.75 U kg^−1^ body weight) after 6–8 h of fasting. Blood samples were drawn from the tail vein before and 15, 30, 60, 90, and 120 min after glucose or insulin injection, and glucose levels were measured using a glucose meter (Roche). Liver triglycerides were measured using a diagnostic kit according to the manufacturer's instructions (Randox Laboratories).

For metabolic cage analysis, mice were fed with HFD for 16 weeks and then housed individually in metabolic chambers (PhenoMaster, TSE Systems) with a 12 h–12 h dark‐light cycle for a period of 72 h. Every 10 min, the volume of oxygen consumption (VO_2_) and carbon dioxide production (VCO_2_) were measured. The respiratory exchange ratio (RER) was defined as VCO_2_/VO_2_, and the energy expenditure (EE) was determined using the following formula;

EE = 3.941 × VO_2_ + 1.106 × VCO_2_. The fat and lean proportions of mice were measured by MRI.

*Oil‐red‐O Staining*: Oil‐red‐O working solution was prepared by adding 6 mL stock (0.5 g Oil‐red‐O in 100 mL isopropanol) to 4 mL distilled water and then filtering through a 0.45 µm filter. Fresh liver tissues were embedded in the optimum cutting temperature embedding agent and then frozen for 24 h. Tissues were cut into 8 µm slices and then were fixed with 4% paraformaldehyde and stained with Oil‐red‐O for 20 min at 37 °C. Afterward, tissue slides were carefully rinsed several times with distilled water to remove excess stains and precipitates. Stained slides were observed using an optical microscope equipped with an imaging system (Olympus).

### Measurements of In Vivo Insulin Signaling

After fasting for 6–8 h, mice were i.p. injected with insulin (2 U Kg^−1^). After 10 min, the mice were euthanized, and their epiWAT and ingWAT were harvested, homogenized, and lysed at 4 °C. Lysates were subjected to western blotting to assess insulin‐stimulated phosphorylation cascades.

### Adipose Tissue Macrophage (ATM) Isolation

EpiWAT from male C57BL/6J mice fed with ND or HFD chow were excised and minced in PBS with calcium chloride and 0.5% BSA. The suspensions were then centrifuged at 500 ×*g* for 5 min to remove erythrocytes and free leukocytes. The pellets were resuspended, and collagenase (1 mg mL^−1^) was added at 37 °C for 30 min with shaking at 150 rpm. After digestion, the cell suspension was filtered through a 100 µm filter and then spun at 300 ×*g* for 5 min to separate floating adipocytes from the SVF pellet. The pellet was resuspended in RBC Lysis Buffer (eBioscience) and incubated for 5 min to remove the erythrocytes. The remaining cells were resuspended in sorting buffer (PBS with 0.5% endotoxin‐free FBS, 2 × 10^−3^ m EDTA, and 25 × 10^−3^ m HEPES) at a concentration of 10^7^ cells mL^−1^. After incubation with Fc Block (BD Biosciences), the cells were stained with conjugated antibodies for 25 min at 4 °C followed by two washes with 0.5% BSA PBS. The cells were then resuspended in sorting buffer supplemented with DAPI and analyzed by FACS (FACSAria; BD Biosciences). Viable cells were analyzed with ATMs identified by coexpression of F4/80 and CD11b.

### Palmitate‐BSA Preparation

Briefly, 100 × 10^−3^ m sodium palmitate (P9767; Sigma Aldrich) was dissolved in sterile water by alternate heating and vortexing after the solution reached 70 °C. The dissolved palmitate solution was immediately combined with serum‐free DMEM containing 5% NEFA‐free BSA, creating a 5 × 10^−3^ m palmitate solution. Before treatment of the cells, the 5 × 10^−3^ m palmitate solution was shaken at 140 rpm at 40 °C for 1 h. Serum‐free DMEM containing 5% NEFA‐free BSA was used as vehicle control.

### Adipocytes Culture Medium (ACM) Collection

ACM was collected from induced‐differentiated adipocytes. In brief, 3 × 10^5^ 3T3‐L1 cells or mouse SVF were cultured in 6‐well plates with DMEM plus 10% FBS. After 100% confluence, cells were cultured in adipogenic media (DMEM with 10% FBS, 0.25 × 10^−3^ m IBMX, 1 mM dexamethasone and 1 µg mL^−1^ insulin). After 2 d, the cells were cultured in adipogenic maintenance media (DMEM with 10% FBS and 1 µg mL^−1^ insulin) for 4 d; the medium was changed every 2 d. Subsequently, the cells were maintained in a normal medium without insulin for 2 d. When about 70–80% fat cells were observed, the culture medium was collected and stored at −20 °C.

### BMDM Cell Culture

To prepare primary bone marrow‐derived macrophages (BMDMs), bone marrow of the tibia and femur of 6‐ to 8‐week‐old CD146 WT and CD146 KO mice was flushed, and the cells were harvested and grown in a complete DMEM culture medium supplemented with 15% L929‐conditioned media for 7 d. In some assays, differentiated BMDMs were stimulated with indicated concentrations of palmitate conjugated with BSA or BSA as a control for 24 h or treated with conditioned medium from differentiated adipocytes. For M1‐like macrophage differentiation, BMDMs were treated with LPS (50 ng mL^−1^) and IFN*γ* (100 ng mL^−1^) for 48 h; for M2‐like macrophage differentiation, BMDMs were treated with IL‐4 (10 ng mL^−1^) for 48 h.

### Cell Line and Plasmids

Raw264.7 was obtained from ATCC. Knockdown of CD146 was performed using CD146 shRNA, which was constructed within the lentiviral vector pHS‐ASR (SyngenTech). The target sequences were as follows: CD146shRNA: GCGGGAACCTGGTGAATATGA, which was reported in a previous study;^[^ ^22^ ^]^ and NC‐shRNA: GTAATTGTCAAATCAGAGTGCT. Knockdown of Gp130 was performed using Gp130 shRNA carried by the lentiviral vector pHS‐ASR (SyngenTech). The target sequences were as follows: Gp130shRNA: CGTCTTGTTCTGCTTTAACAA; and NC‐shRNA: AAACGTGACACGTTCGGAGAA.

### RT‐qPCR

Total RNA was isolated using TRIzol reagent (Invitrogen), reverse transcribed into cDNA using random primers, and then subjected to qPCR using ChamQ Universal SYBR qPCR Master reagent (Vazyme Biotech Co., Ltd, China) according to the manufacturer's instructions. The primers used are listed in Table S1 (Supporting Information). *Rpl13a* was used as the housekeeping gene. The expression level of each gene was normalized as described in the corresponding figure legend.

### Co‐Immunoprecipitation and Western Blotting

Raw264.7 cells were transfected with plasmids encoding full‐length or truncated CD146 with 3×Flag tag at the C‐terminal end using Lipofectamine 2000. Transfected Raw264.7 cells were lysed in ice‐cold RIPA lysis buffer (150 × 10^−3^ m NaCl, 50 × 10^−3^ m Tris, pH 7.4, 0.25% sodium deoxycholate, 1% NP‐40, 1 × 10^−3^ m PMSF, protease inhibitor cocktails) for 1 h. After centrifugation at 12 000 × g for 10 min, the supernatant was immunoprecipitated with anti‐FLAG M2 agarose beads (Sigma) overnight at 4 °C. The immunoprecipitants were washed three times with a washing buffer (1 m NaCl, 1% NP‐40, 50 × 10^−3^ m Tris–HCl, pH 7.5) and then incubated with 200 µL Elution buffer (washing buffer, 200 × 10^−3^ m Tris–HCl, 0.2 mg mL^−1^ flag peptide) for 1 h at 4 °C. The supernatant was collected and analyzed by western blotting. Clinx ChemiCapture (China) was used for protein detection, and Clinx image analysis software was used for protein gray value analysis.

### Pull‐Down Assay

Purified mouse Fc‐CD146 and Gp130 protein (1 µg each) were incubated in PBS for 1 h at 4 °C. For the antibody blocking assay, Fc‐CD146 was first incubated with AA98 or mIgG (0.5 µg) for 30 min, and then Gp130 protein was added for 1 h at 4 °C. After immunoprecipitation with protein G PLUS‐agarose (Santa Cruz Biotechnology), Fc tag and Gp130 were measured by western blotting.

### Labeling and Tracking of Macrophages

A macrophage tracking technique was used to specifically examine the role of CD146 in macrophage emigration.^[^ ^19^ ^]^ Briefly, 5×10^6^ bone marrow cells from M‐WT or M‐KO donor mice were transferred to lethally irradiated CD45.1^+^ CD146 WT host mice that had been fed with HFD for 12 weeks. After 8 weeks, at which time the reconstitution was completed, mice were injected intravenously with 200 µL microspheres (YG‐beads) diluted in sterile PBS (1:4). The labeling efficiency (percentage of bead‐positive blood monocytes) was measured by flow cytometry at the indicated days after the beads were injected.

### Statistical Analyses

All experiments were performed independently at least three times. Bar graphs show the mean ± SEM or SD, as indicated in each legend. Differences between groups were examined by Student's *t*‐test or one‐way ANOVA followed by Bonferroni's correction or two‐way ANOVA with the multiple‐comparison test for comparison of means. *p*‐values of less than 0.05 were considered statistically significant. GraphPad Prism software was used for calculations (GraphPad Inc., San Diego, CA, USA).

## Conflict of Interest

The authors declare no conflict of interest.

## Author Contributions

H.D. and L.J. contributed equally to this work. H.D. and L.J. designed and performed experiments, analyzed data, and wrote the manuscript; Z.W., J.X., J.L., X.M., and Z.L. assisted with experiments; X.C. and J.F. analyzed the data; X.Y. designed the research and wrote the manuscript.

## Supporting information

Supporting Information

## Data Availability

The data that support the findings of this study are available from the corresponding author upon reasonable request.

## References

[1] M. C. Petersen , G. I. Shulman , Physiol. Rev. 2018, 98, 2133. 30067154 10.1152/physrev.00063.2017 PMC6170977

[2] J. C. McNelis , J. M. Olefsky , Immunity 2014, 41, 36. 25035952 10.1016/j.immuni.2014.05.010

[3] S. P. Weisberg , D. McCann , M. Desai , M. Rosenbaum , R. L. Leibel , A. W. Ferrante Jr. , J. Clin. Invest. 2003, 112, 1796. 14679176 10.1172/JCI19246 PMC296995

[4] Y. S. Lee , J. Wollam , J. M. Olefsky , Cell 2018, 172, 22. 29328913 10.1016/j.cell.2017.12.025 PMC8451723

[5] F. Zatterale , M. Longo , J. Naderi , G. A. Raciti , A. Desiderio , C. Miele , F. Beguinot , Front. Physiol. 2019, 10, 1607. 32063863 10.3389/fphys.2019.01607 PMC7000657

[6] C. N. Lumeng , J. L. Bodzin , A. R. Saltiel , J. Clin. Invest. 2007, 117, 175. 17200717 10.1172/JCI29881 PMC1716210

[7] M. Kratz , B. R. Coats , K. B. Hisert , D. Hagman , V. Mutskov , E. Peris , K. Q. Schoenfelt , J. N. Kuzma , I. Larson , P. S. Billing , R. W. Landerholm , M. Crouthamel , D. Gozal , S. Hwang , P. K. Singh , L. Becker , Cell Metab. 2014, 20, 614. 25242226 10.1016/j.cmet.2014.08.010 PMC4192131

[8] M. Zeyda , K. Gollinger , E. Kriehuber , F. W. Kiefer , A. Neuhofer , T. M. Stulnig , Int. J. Obes. 2010, 34, 1684. 10.1038/ijo.2010.103 20514049

[9] J. M. Wentworth , G. Naselli , W. A. Brown , L. Doyle , B. Phipson , G. K. Smyth , M. Wabitsch , P. E. O'Brien , L. C. Harrison , Diabetes 2010, 59, 1648. 20357360 10.2337/db09-0287 PMC2889764

[10] L. Boutens , R. Stienstra , Diabetologia 2016, 59, 879. 26940592 10.1007/s00125-016-3904-9 PMC4826424

[11] L. Russo , C. N. Lumeng , Immunology 2018, 155, 407. 30229891 10.1111/imm.13002 PMC6230999

[12] L. Cron , T. Allen , M. A. Febbraio , J. Exp. Biol. 2016, 219, 259. 26792338 10.1242/jeb.129213

[13] M. A. Febbraio , J. Clin. Invest. 2007, 117, 841. 17404609 10.1172/JCI30453 PMC1838942

[14] M. J. Kraakman , T. L. Allen , M. Whitham , P. Iliades , H. L. Kammoun , E. Estevez , G. I. Lancaster , M. A. Febbraio , Diabetes, Obes. Metab. 2013, 15, 170. 24003934 10.1111/dom.12170

[15] V. Wallenius , K. Wallenius , B. Ahren , M. Rudling , H. Carlsten , S. L. Dickson , C. Ohlsson , J. O. Jansson , Nat. Med. 2002, 8, 75. 11786910 10.1038/nm0102-75

[16] J. Mauer , B. Chaurasia , J. Goldau , M. C. Vogt , J. Ruud , K. D. Nguyen , S. Theurich , A. C. Hausen , J. Schmitz , H. S. Bronneke , E. Estevez , T. L. Allen , A. Mesaros , L. Partridge , M. A. Febbraio , A. Chawla , F. T. Wunderlich , J. C. Bruning , Nat. Immunol. 2014, 15, 423. 24681566 10.1038/ni.2865 PMC4161471

[17] Z. Wang , X. Yan , Cancer Lett. 2013, 330, 150. 23266426 10.1016/j.canlet.2012.11.049

[18] P. K. Dagur , J. P. McCoy Jr. , Autoimmun. Rev. 2015, 14, 415. 25595133 10.1016/j.autrev.2015.01.003 PMC4369459

[19] Y. Luo , H. Duan , Y. Qian , L. Feng , Z. Wu , F. Wang , J. Feng , D. Yang , Z. Qin , X. Yan , Cell Res. 2017, 27, 352. 28084332 10.1038/cr.2017.8 PMC5339843

[20] H. Duan , S. Xing , Y. Luo , L. Feng , I. Gramaglia , Y. Zhang , D. Lu , Q. Zeng , K. Fan , J. Feng , D. Yang , Z. Qin , P. O. Couraud , I. A. Romero , B. Weksler , X. Yan , Sci. Rep. 2013, 3, 1687. 23595028 10.1038/srep01687 PMC3629416

[21] S. Xing , Y. Luo , Z. Liu , P. Bu , H. Duan , D. Liu , P. Wang , J. Yang , L. Song , J. Feng , D. Yang , Z. Qin , X. Yan , Am J Pathol 2014, 184, 1604. 24767106 10.1016/j.ajpath.2014.01.031

[22] H. Duan , S. Zhao , J. Xiang , C. Ju , X. Chen , I. Gramaglia , X. Yan , Cell Mol. Immunol. 2021, *18*, 2443. 33203936 10.1038/s41423-020-00582-8 PMC8484550

[23] M. Furuhashi , R. Fucho , C. Z. Gorgun , G. Tuncman , H. Cao , G. S. Hotamisligil , J. Clin. Invest. 2008, 118, 2640. 18551191 10.1172/JCI34750 PMC2423863

[24] I. R. Klein‐Wieringa , S. N. Andersen , J. C. Kwekkeboom , M. Giera , B. J. de Lange‐Brokaar , G. J. van Osch , A. M. Zuurmond , V. Stojanovic‐Susulic , R. G. Nelissen , H. Pijl , T. W. Huizinga , M. Kloppenburg , R. E. Toes , A. Ioan‐Facsinay , J. Immunol. 2013, 191, 1356. 23817431 10.4049/jimmunol.1203074

[25] S. Wueest , R. A. Rapold , J. M. Rytka , E. J. Schoenle , D. Konrad , Diabetologia 2009, 52, 541. 19048227 10.1007/s00125-008-1223-5

[26] S. Nielsen , Z. Guo , C. M. Johnson , D. D. Hensrud , M. D. Jensen , J. Clin. Invest. 2004, 113, 1582. 15173884 10.1172/JCI21047 PMC419492

[27] K. J. Chung , A. Chatzigeorgiou , M. Economopoulou , R. Garcia‐Martin , V. I. Alexaki , I. Mitroulis , M. Nati , J. Gebler , T. Ziemssen , S. E. Goelz , J. Phieler , J. H. Lim , K. P. Karalis , T. Papayannopoulou , M. Bluher , G. Hajishengallis , T. Chavakis , Nat. Immunol. 2017, 18, 654. 28414311 10.1038/ni.3728 PMC5436941

[28] J. M. van Gils , M. C. Derby , L. R. Fernandes , B. Ramkhelawon , T. D. Ray , K. J. Rayner , S. Parathath , E. Distel , J. L. Feig , J. I. Alvarez‐Leite , A. J. Rayner , T. O. McDonald , K. D. O'Brien , L. M. Stuart , E. A. Fisher , A. Lacy‐Hulbert , K. J. Moore , Nat. Immunol. 2012, 13, 136. 22231519 10.1038/ni.2205 PMC3262880

[29] U. A. White , J. M. Stephens , Curr. Pharm. Des. 2011, 17, 340. 21375496 10.2174/138161211795164202 PMC3119891

[30] C. Zheng , Y. Qiu , Q. Zeng , Y. Zhang , D. Lu , D. Yang , J. Feng , X. Yan , Int. J. Biochem. Cell Biol. 2009, 41, 2163. 19782948 10.1016/j.biocel.2009.03.014

[31] J. A. B. Pedroso , A. M. Ramos‐Lobo , J. Donato Jr. , Hormones 2019, 18, 127. 30414080 10.1007/s42000-018-0078-5

[32] C. D. Chung , J. Liao , B. Liu , X. Rao , P. Jay , P. Berta , K. Shuai , Science 1997, 278, 1803. 9388184 10.1126/science.278.5344.1803

[33] H. Qin , K. L. Roberts , S. A. Niyongere , Y. Cong , C. O. Elson , E. N. Benveniste , J. Immunol. 2007, 179, 5966. 17947670 10.4049/jimmunol.179.9.5966

[34] A. A. Hill , W. R. Bolus , A. H. Hasty , Immunol. Rev. 2014, 262, 134. 25319332 10.1111/imr.12216 PMC4203421

[35] X. Liao , N. Sharma , F. Kapadia , G. Zhou , Y. Lu , H. Hong , K. Paruchuri , G. H. Mahabeleshwar , E. Dalmas , N. Venteclef , C. A. Flask , J. Kim , B. W. Doreian , K. Q. Lu , K. H. Kaestner , A. Hamik , K. Clement , M. K. Jain , J. Clin. Invest. 2011, 121, 2736. 21670502 10.1172/JCI45444 PMC3223832

[36] J. I. Odegaard , R. R. Ricardo‐Gonzalez , M. H. Goforth , C. R. Morel , V. Subramanian , L. Mukundan , A. Red Eagle , D. Vats , F. Brombacher , A. W. Ferrante , A. Chawla , Nature 2007, 447, 1116. 17515919 10.1038/nature05894 PMC2587297

[37] F. Villarroya , R. Cereijo , J. Villarroya , A. Gavalda‐Navarro , M. Giralt , Cell Metab. 2018, 27, 954. 29719233 10.1016/j.cmet.2018.04.006

[38] N. Cox , L. Crozet , I. R. Holtman , P. L. Loyher , T. Lazarov , J. B. White , E. Mass , E. R. Stanley , O. Elemento , C. K. Glass , F. Geissmann , Science 2021, 373, eabe9383. 10.1126/science.abe9383 PMC9558257 34210853

[39] C. J. O. O'Brien , A. Domingos , Science 2021, 373, 24.34210864

[40] Q. Wu , S. R. Case , M. N. Minor , D. Jiang , R. J. Martin , R. P. Bowler , J. Wang , J. Hartney , A. Karimpour‐Fard , H. W. Chu , Am J Pathol 2013, 182, 819. 23256918 10.1016/j.ajpath.2012.11.005 PMC3586690

[41] T. Suganami , K. Tanimoto‐Koyama , J. Nishida , M. Itoh , X. Yuan , S. Mizuarai , H. Kotani , S. Yamaoka , K. Miyake , S. Aoe , Y. Kamei , Y. Ogawa , Arterioscler., Thromb., Vasc. Biol. 2007, 27, 84. 17082484 10.1161/01.ATV.0000251608.09329.9a

[42] D. Patsouris , P. P. Li , D. Thapar , J. Chapman , J. M. Olefsky , J. G. Neels , Cell Metab. 2008, 8, 301. 18840360 10.1016/j.cmet.2008.08.015 PMC2630775

[43] O. Patsalos , B. Dalton , J. Leppanen , M. A. A. Ibrahim , H. Himmerich , Front. Pharmacol. 2020, 11, 481. 32351392 10.3389/fphar.2020.00481 PMC7174757

[44] C. D. Camell , J. Sander , O. Spadaro , A. Lee , K. Y. Nguyen , A. Wing , E. L. Goldberg , Y. H. Youm , C. W. Brown , J. Elsworth , M. S. Rodeheffer , J. L. Schultze , V. D. Dixit , Nature 2017, 550, 119. 28953873 10.1038/nature24022 PMC5718149

[45] R. M. Pirzgalska , E. Seixas , J. S. Seidman , V. M. Link , N. M. Sanchez , I. Mahu , R. Mendes , V. Gres , N. Kubasova , I. Morris , B. A. Arus , C. M. Larabee , M. Vasques , F. Tortosa , A. L. Sousa , S. Anandan , E. Tranfield , M. K. Hahn , M. Iannacone , N. J. Spann , C. K. Glass , A. I. Domingos , Nat. Med. 2017, 23, 1309. 29035364 10.1038/nm.4422 PMC7104364

[46] N. N. Pejnovic , J. M. Pantic , I. P. Jovanovic , G. D. Radosavljevic , M. Z. Milovanovic , I. G. Nikolic , N. S. Zdravkovic , A. L. Djukic , N. N. Arsenijevic , M. L. Lukic , Diabetes 2013, 62, 1932. 23349493 10.2337/db12-0222 PMC3661611

[47] V. Serbulea , C. M. Upchurch , M. S. Schappe , P. Voigt , D. E. DeWeese , B. N. Desai , A. K. Meher , N. Leitinger , Proc. Natl. Acad. Sci. USA 2018, 115, E6254. 29891687 10.1073/pnas.1800544115 PMC6142199

[48] S. D. Sackett , T. Otto , A. Mohs , L. E. Sander , S. Strauch , K. L. Streetz , D. C. Kroy , C. Trautwein , FASEB J. 2019, 33, 6035. 30726111 10.1096/fj.201802118R

[49] A. Matsukawa , S. Kudo , T. Maeda , K. Numata , H. Watanabe , K. Takeda , S. Akira , T. Ito , J. Immunol. 2005, 175, 3354. 16116228 10.4049/jimmunol.175.5.3354

[50] Z. Yin , T. Ma , Y. Lin , X. Lu , C. Zhang , S. Chen , Z. Jian , J. Cell. Biochem. 2018, 119, 9419. 30015355 10.1002/jcb.27259

[51] R. Weiss , J. Dziura , T. S. Burgert , W. V. Tamborlane , S. E. Taksali , C. W. Yeckel , K. Allen , M. Lopes , M. Savoye , J. Morrison , R. S. Sherwin , S. Caprio , N. Engl. J. Med. 2004, 350, 2362. 15175438 10.1056/NEJMoa031049

[52] H. J. Kim , T. Higashimori , S. Y. Park , H. Choi , J. Dong , Y. J. Kim , H. L. Noh , Y. R. Cho , G. Cline , Y. B. Kim , J. K. Kim , Diabetes 2004, 53, 1060. 15047622 10.2337/diabetes.53.4.1060

[53] C. Garbers , S. Heink , T. Korn , S. Rose‐John , Nat. Rev. Drug Discovery 2018, 17, 395. 29725131 10.1038/nrd.2018.45

[54] G. E. Diehl , R. S. Longman , J. X. Zhang , B. Breart , C. Galan , A. Cuesta , S. R. Schwab , D. R. Littman , Nature 2013, 494, 116. 23334413 10.1038/nature11809 PMC3711636

[55] B. Ramkhelawon , E. J. Hennessy , M. Menager , T. D. Ray , F. J. Sheedy , S. Hutchison , A. Wanschel , S. Oldebeken , M. Geoffrion , W. Spiro , G. Miller , R. McPherson , K. J. Rayner , K. J. Moore , Nat. Med. 2014, 20, 377. 24584118 10.1038/nm.3467 PMC3981930

[56] M. Sharma , M. Schlegel , E. J. Brown , B. E. Sansbury , A. Weinstock , M. S. Afonso , E. M. Corr , C. van Solingen , L. C. Shanley , D. Peled , R. Ramasamy , A. M. Schmidt , M. Spite , E. A. Fisher , K. J. Moore , Immunometabolism. 2019, 1, 190010. 10.20900/immunometab20190010 PMC6699780 31428465

[57] L. Love‐Gregory , N. A. Abumrad , Curr. Opin. Clin. Nutr. Metab. Care 2011, 14, 527. 21912245 10.1097/MCO.0b013e32834bbac9 PMC3295590

[58] D. J. Kennedy , S. Kuchibhotla , K. M. Westfall , R. L. Silverstein , R. E. Morton , M. Febbraio , Cardiovasc. Res. 2011, 89, 604. 21088116 10.1093/cvr/cvq360 PMC3028977

[59] K. Miyaoka , T. Kuwasako , K. Hirano , S. Nozaki , S. Yamashita , Y. Matsuzawa , Lancet 2001, 357, 686. 11247555 10.1016/s0140-6736(00)04138-6

[60] T. Kuwasako , K. Hirano , N. Sakai , M. Ishigami , H. Hiraoka , M. J. Yakub , K. Yamauchi‐Takihara , S. Yamashita , Y. Matsuzawa , Diabetes Care 2003, 26, 1647. 10.2337/diacare.26.5.1647-a 12716848

[61] J. Smith , X. Su , R. El‐Maghrabi , P. D. Stahl , N. A. Abumrad , J. Biol. Chem. 2008, 283, 13578. 18353783 10.1074/jbc.M800008200 PMC2376227

[62] Z. Wu , J. Liu , G. Chen , J. Du , H. Cai , X. Chen , G. Ye , Y. Luo , Y. Luo , L. Zhang , H. Duan , Z. Liu , S. Yang , H. Sun , Y. Cui , L. Sun , H. Zhang , G. Shi , T. Wei , P. Liu , X. Yan , J. Feng , P. Bu , Adv. Sci. 2021, 8, 2004032. 10.1002/advs.202004032 PMC7967059 33747748

[63] X. Yan , Y. Lin , D. Yang , Y. Shen , M. Yuan , Z. Zhang , P. Li , H. Xia , L. Li , D. Luo , Q. Liu , K. Mann , B. L. Bader , Blood 2003, 102, 184. 12609848 10.1182/blood-2002-04-1004

[64] H. Duan , S. Zhao , J. Xiang , C. Ju , X. Chen , I. Gramaglia , X. Yan , Cell Mol. Immunol. 2021, 18, 2443. 33203936 10.1038/s41423-020-00582-8 PMC8484550

[65] Y. Zhang , C. Zheng , J. Zhang , D. Yang , J. Feng , D. Lu , X. Yan , Hybridoma 2008, 27, 345. 18847347 10.1089/hyb.2008.0034

