# Supplementary material for: CD146 Associates with Gp130 to Control a Macrophage Pro‐inflammatory Program That Regulates the Metabolic Response to Obesity
Source: Adv Sci (Weinh). 2022 Mar 8;9(13):2103719. doi: 10.1002/advs.202103719 (PMC9069186; doi:10.1002/advs.202103719)
Supplement: Supplementary file 1 — Supporting Information [file ADVS-9-2103719-s001.pdf]

Supporting Information

CD146 Associates with Gp130 to Control a Macrophage Pro-inflammatory Program that Regulates the Metabolic Response to Obesity

Hongxia Duan, Lin Jing, Jianquan Xiang, Chenhui Ju, Zhenzhen Wu, Jingyu Liu, Xinran Ma, Xuehui Chen, Zheng Liu, Jing Feng, Xiyun Yan**

Supplementary Figures

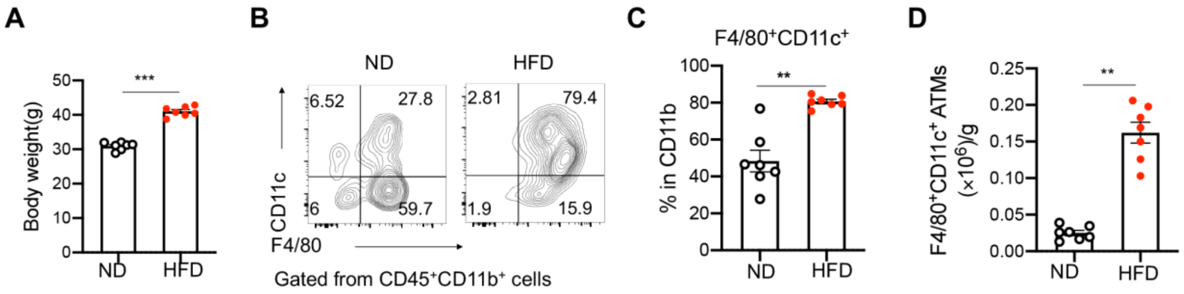

Figure S1. Accumulation of macrophages in adipose tissue after diet-induced obesity. A) Body weights of C57 mice fed with ND or HFD for 12 weeks (n = 7). B) Surface staining of F4/80 and CD11c on $CD45^+CD11b^+$ cells from ND- or HFD-fed mice (representative of n = 3). The numbers in the outlined areas indicate the percentages for each gate. C, D) Percentages (C) and numbers (D) of $CD11c^+F4/80^+$ cells in the $CD11b^+$ cells from ND and HFD mice (n = 7). Each symbol (A, C and D) represents an individual mouse; the short horizontal lines indicate the mean ± SEM. For statistical significance, a two-tailed t-test (A, C, and D) was performed. $^{**}p<0.01$, $^{***}p<0.001$.

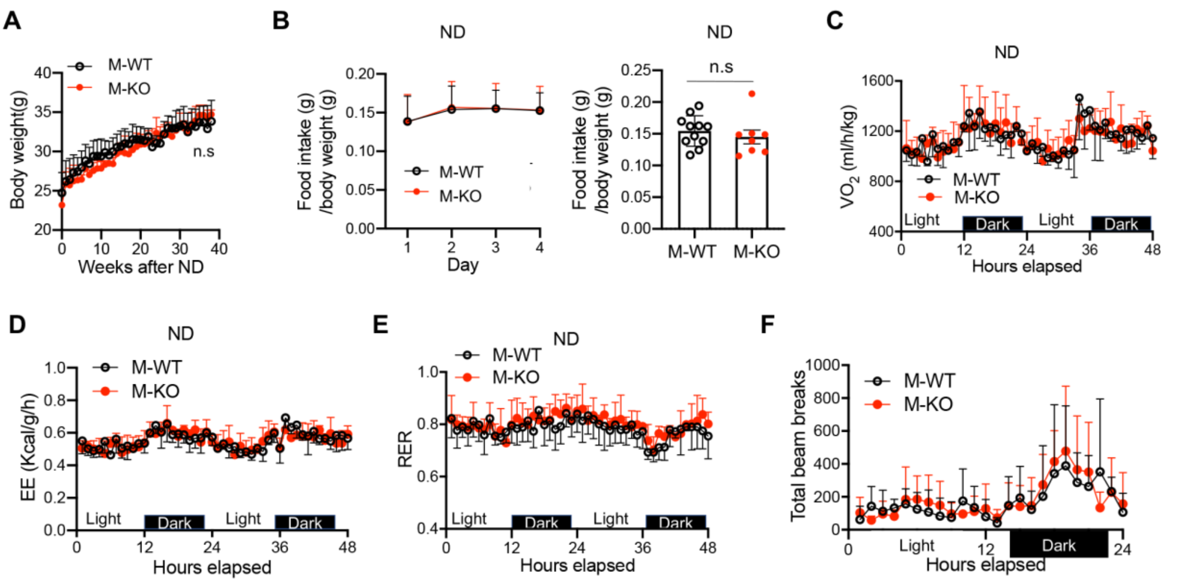

Figure S2. Macrophage CD146 does not affect the metabolism of ND mice. A) Weight gains of M-KO and M-WT mice fed with chow diet (ND) (n = 7). B) Food intake analysis of ND mice (n = 11 for M-WT or 8 for M-KO). C–E) Metabolic cage analysis of VO_2, energy expenditure and RER of ND mice (n = 5). F) Physical activity of HFD mice (n = 5). Each symbol (B) represents an individual mouse; the short horizontal lines indicate the mean ± SEM. Two-way ANOVA with multiple-comparison test (A, B, C, D, E, and F) or two-tailed t-test (B) was performed to test statistical significance. ns, not significant.

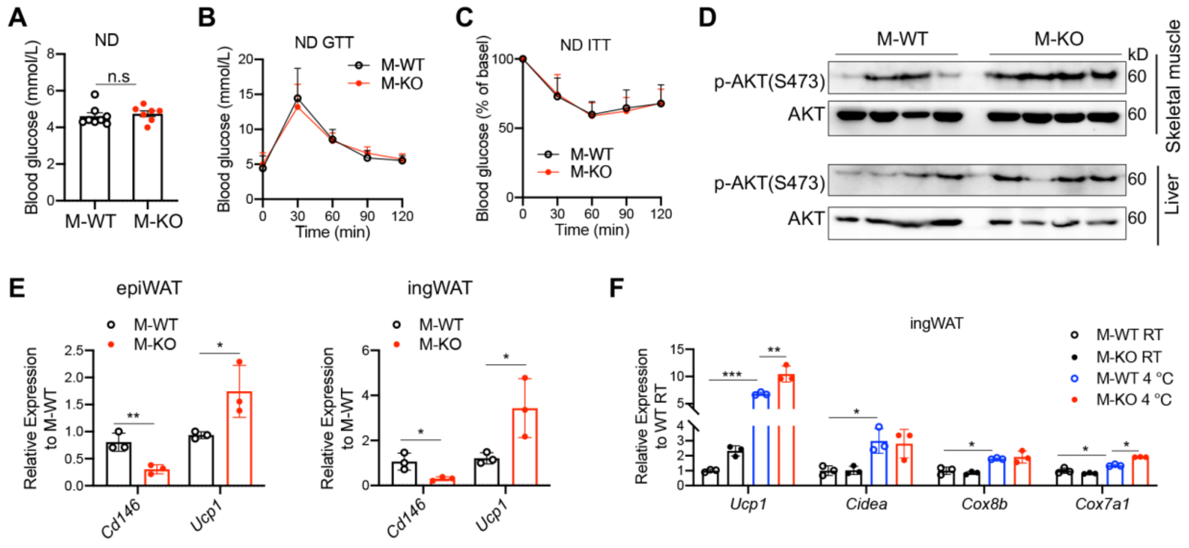

Figure S3. Deficiency of CD146 on macrophages improves insulin sensitivity. A-C) Fasting blood concentrations of glucose (A), GTT (B) and ITT (C) in M-WT and M-KO mice fed with a normal diet (ND) (*n* = 7). D) Immunoblot analyses of skeletal muscle and liver tissue samples were performed using antibodies specific to Akt and phospho-Akt (pAKT-S473) (representative of n = 3). E) Analysis of mRNA levels (*Cd146* and *Ucp1*) from adipose tissue (n = 3). F) Analysis of thermogenesis genes in ingWAT from mice kept at room temperature (RT) or 4℃ for 72 h (n = 3). Each symbol (A, E, and F) represents an individual mouse; the short horizontal lines indicate the mean ± SEM (A,B and C) or SD (E and F). Two-tailed t-test (A) or 2-way ANOVA with multiple-comparison test (B and C) or one-way ANOVA (E and F was performed to test statistical significance. n.s, not significant. *p<0.05, **p<0.01, ***p<0.001.

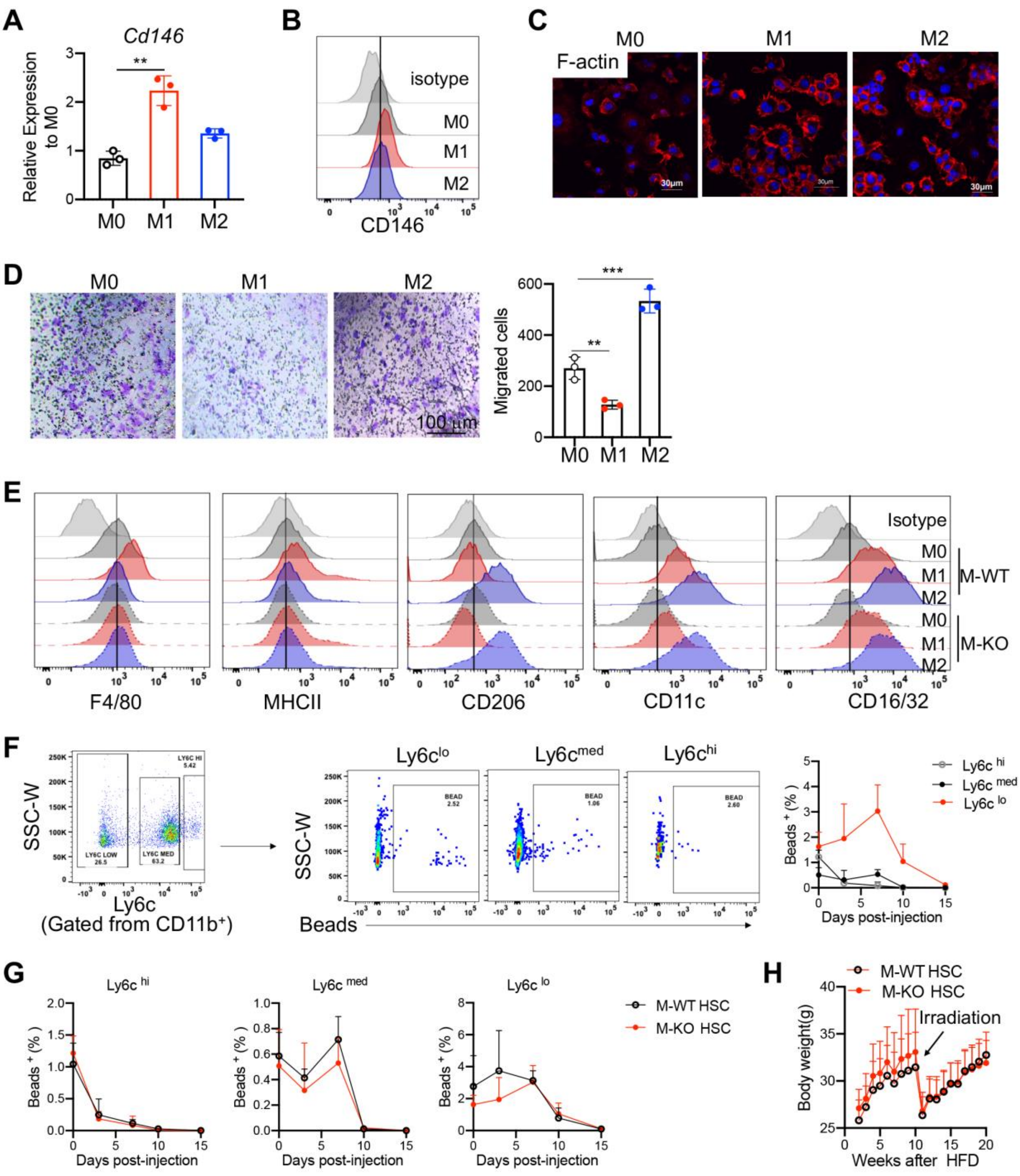

Figure S4. Macrophage deficiency of CD146 reduces macrophage retention in the adipose tissue. A) Relative CD146 gene expression in M0, M1 and M2 macrophages (n = 3). B) Surface staining of CD146 on M0, M1 and M2 macrophages (representative of n = 3). C) Immunofluorescence of F-actin (red) and nuclear (blue) staining in M0, M1 and M2 macrophages (representative of n = 3). Scale bar, 30 μm. D) Migrated M0, M1 and M2 macrophages in sera (representative of n = 3). Scale bar, 100 μm. E) Surface staining of F4/80, MHCII, CD206, CD11c and CD16/32 in M0, M1 and M2 cells induced from M-WT or M-KO BMDMs (representative of n = 3). F) Dynamic change of percentages of bead-labeled myeloid cells in the blood in WT mice (*n* = 5). G) Dynamic change of percentages of bead-labeled myeloid cells in the blood of M-WT HSC and M-KO HSC mice (*n* = 5). H) Body weights of M-WT HSC and M-KO HSC mice (*n* = 5). Each symbol (A, D) represents an individual experiment. The short horizontal lines indicate the mean ± SD (A

and D) or SEM (F,G and H). One-way ANOVA (A, D) was performed to test statistical significance. **p<0.01, ***p<0.001.

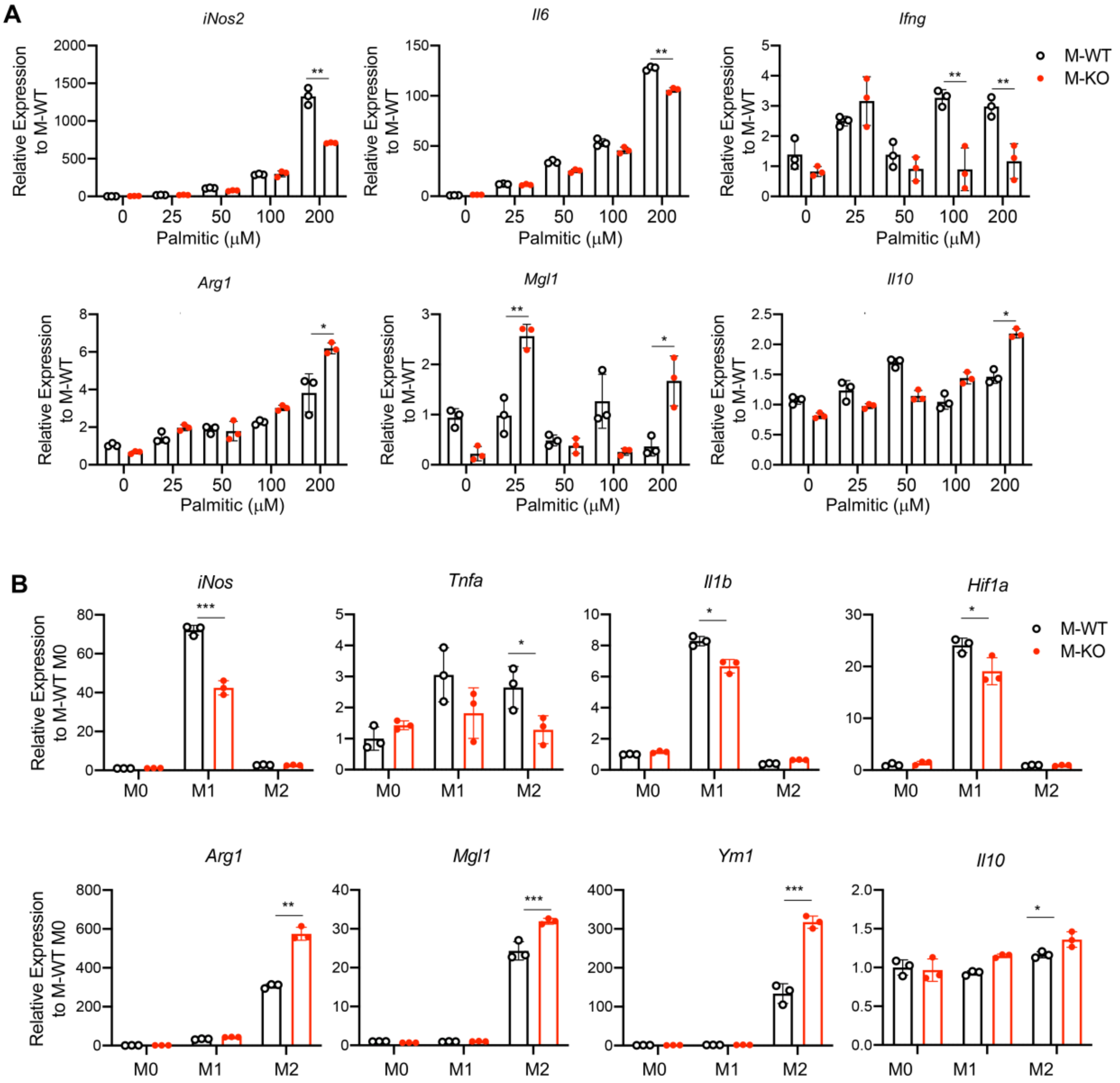

Figure S5. Macrophage deficiency of CD146 mitigates pro-inflammatory polarization. A) Relative gene expression in BMDMs after stimulation with the indicated concentrations of palmitic acid for 36 h (n = 3). B) Relative gene expression in M0, M1 and M2 induced from M-WT or M-KO BMDMs. Each symbol represents an individual experiment. The short horizontal lines indicate the mean ± SD. One-way ANOVA (A and B) was performed to test statistical significance. *p<0.05, **p<0.01, ***p<0.001.

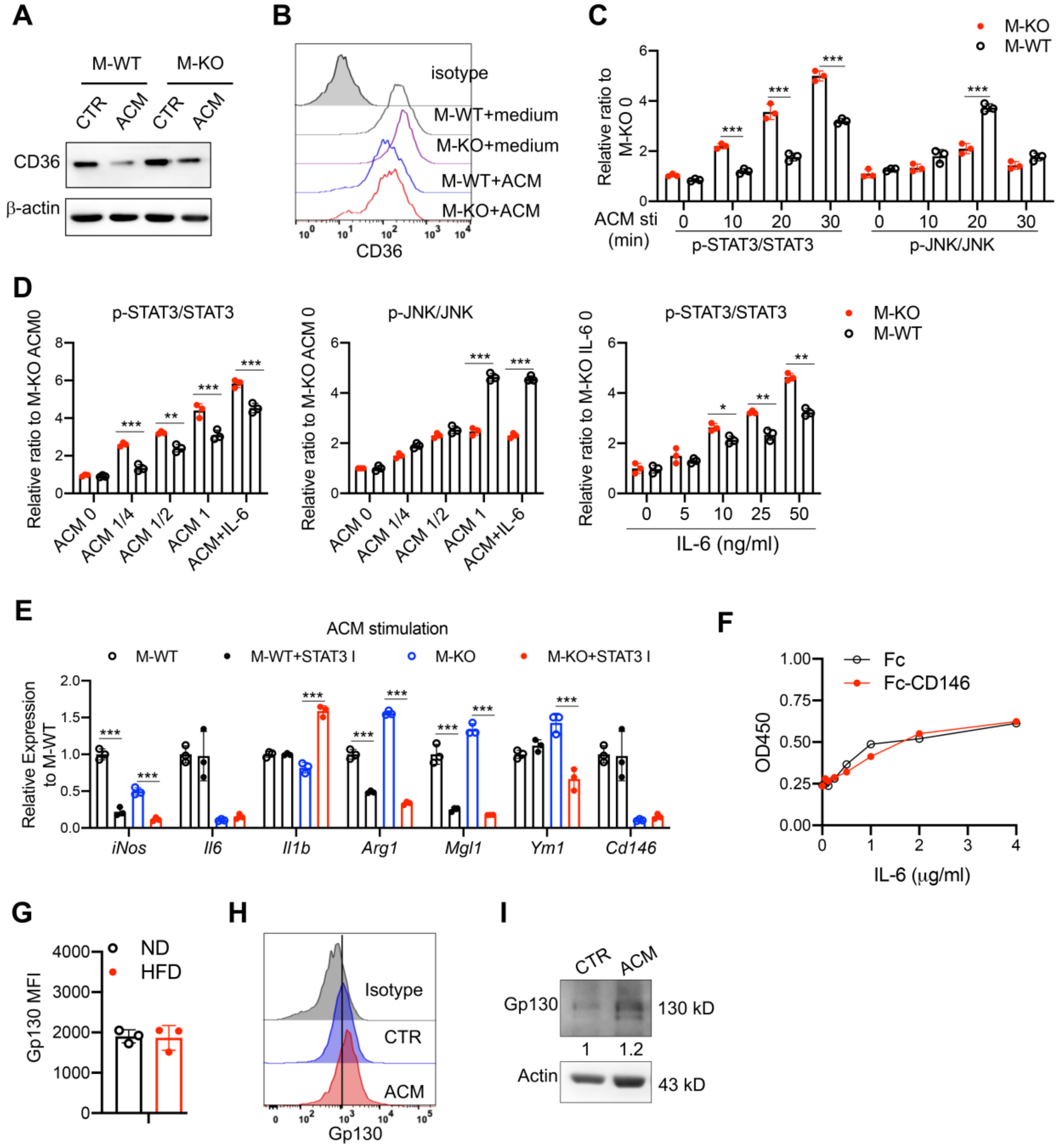

Figure S6. CD146 interacts with Gp130 and negatively regulates the STAT3 activation. A) Immunoblot analyses of BMDMs stimulated with ACM for 24 h using antibodies specific to CD36 (representative of n = 3). β-actin served as a loading control. B) Cell surface staining of CD36 on BMDMs stimulated with or without ACM (representative of n = 3). C) Quantitative analysis of the p-STAT3 / STAT3 ratio of macrophages under ACM stimuli for indicated time (n = 3). D) Quantitative analysis of the ratio of p-STAT3/STAT3, p-JNK/JNK in macrophages under various stimuli (n = 3). E) Relative gene expression in BMDMs after stimulation with ACM plus with or without STAT3 inhibitor for 24 h (n = 3). F) Fc-CD146 or Fc protein was added to wells coated with different concentrations of human IL-6 recombinant protein, and ELISA was performed (representative of n = 3). G) Gp130 expression on ATMs from ND- or HFD-fed mice (n = 3). H) Surface staining of Gp130 on BMDMs stimulated with ACM or control medium (representative of n = 3). I) Immunoblot analyses of Gp130 protein in BMDMs treated with ACM or control medium (representative of n = 3). Actin was used as the loading control. Each symbol represents an individual

experiment (C, D, and E) or an individual mouse (G). The short horizontal lines indicate the mean ± SD. One-way ANOVA (C, D, and E) was performed. *p<0.05, **p<0.01, ***p<0.001.

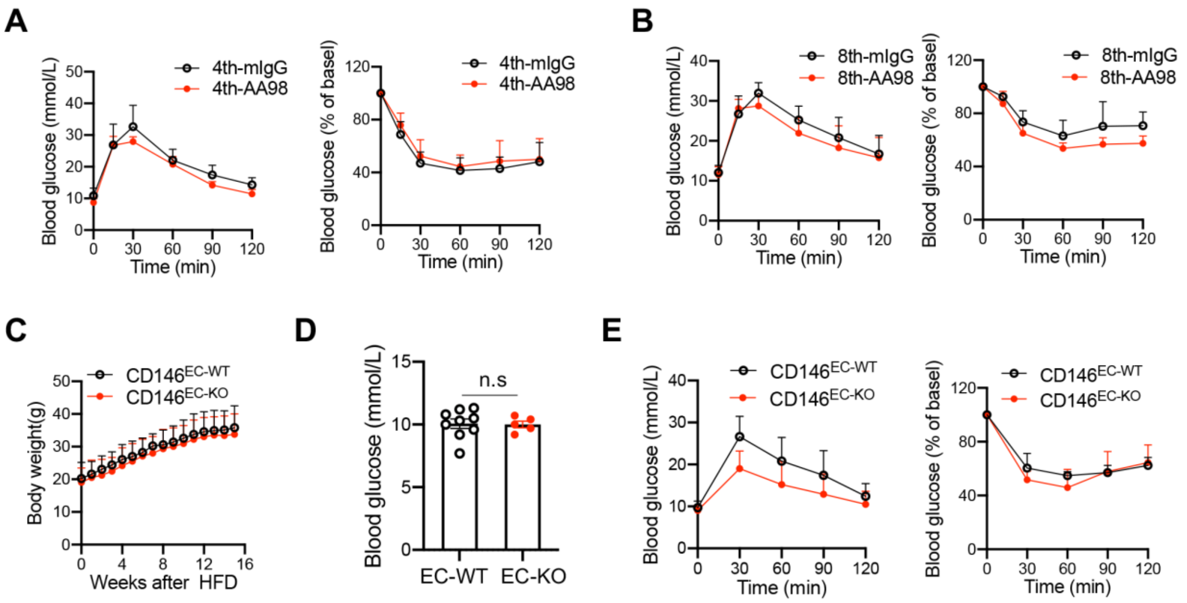

Figure S7. Targeting CD146 with functional antibody AA98 inhibits VAT inflammation in diet-induced obesity. A,B) GTT and ITT of HFD mice treated with antibody for four weeks (A) or eight weeks (B) (*n* = 7). C) Body weights of HFD endothelial CD146 conditional knockout mice (CD146$^{EC\text{-}KO}$, *n* = 5) and their control mice (CD146 $^{EC\text{-}WT}$,*n* = 9). D) Fasting blood glucose of HFD CD146 $^{EC\text{-}WT}$ (*n* = 9) and $^{EC\text{-}KO}$ mice (*n* = 5). E) GTT and ITT of HFD CD146 $^{EC\text{-}WT}$ (*n* = 9) and $^{EC\text{-}KO}$ mice (*n* = 5). Each symbol represents an individual mouse (D); the short horizontal lines indicate the mean ± SEM. Two-way ANOVA with multiple-comparison test (A, B, C, and E) or two-tailed t-test (D) was performed. n.s, no significant difference.

Supplementary Table

Table S1. Primers used in this study

Gene Name	Forward (5' to 3')	Reverse (5' to 3')
mCD146	AGTCCTCACACCAGAGCCAA	CTCTTACGAGTCGGGGGCA
mMgl1	CAGAATCGCTTAGCCAATGTGG	TCCCAGTCCGTGTCCGAAC
mArg1	CCACAGTCTGGCAGTTGGAAG	GGTTGTCAGGGGAGTGTTGATG
miNos	GCGCTCTAGTGAAGCAAAGC	GGCCTTGTGGTGAAGAGTGT
mIl10	GCTCCAAGACCAAGGTGTCT	CGGAGAGAGGTACAAACGAGG
mIl1b	AACAGGCTGCTCTGGGATTC	AGATTCGTAGCTGGATGCCG
mTnfa	CCCAGGGACCTCTCTCTAATCA	AGCTGCCCCTCAGCTTGAG
mIfng	ACGCTACACACTGCATCTTG	GTCACCATCCTTTTGCCAGTTC
mIl6	GCCTTCTTGGGACTGATGCT	CTGCAAGTGCATCATCGTTGT
mHif1a	CACCGATTCGCCATGGA	TTTCTTTTCGACGTTCAGAACTCAT
mUcp1	CGTACCAAGCTGTGCGATGT	GAAGCCACAAACCCTTTGAAAA
mRpl13a	GAGGTCGGGTGGAAGTACCA	TGCATCTTGGCCTTTTCCT
mSocs3	TGAGCGTCAAGACCCAGTCG	CACAGTCGAAGCGGGGAACT
mPias3	CGATGTCTCAAGATGGCGGA	GGAAAGCGCCTGCGATAAAG
mGp130	TACCATGGAGCACCTCCCTT	CACGAAAGAATACGAGCCCA
mCidea	TGACATTCATGGGATTGCAGAC	GGCCAGTTGTGATGACTAAGAC
mDio2	GAAGGCTGCCGAATGTCAAC	GCGTGAGCAGTCTTGTTTGG
mCox8b	TGTGGGGATCTCAGCCATAGT	AGTGGGCTAAGACCCATCCTG
mCox7a1	CAGCGTCATGGTCAGTCTGT	AGAAAACCGTGTGGCAGAGA
